# Single-cell deconvolution algorithms analysis unveils autocrine IL11-mediated resistance to docetaxel in prostate cancer via activation of the JAK1/STAT4 pathway

**DOI:** 10.1186/s13046-024-02962-8

**Published:** 2024-03-01

**Authors:** Bisheng Cheng, Lingfeng Li, Tianlong Luo, Qiong Wang, Yong Luo, Shoumin Bai, Kaiwen Li, Yiming Lai, Hai Huang

**Affiliations:** 1 Department of Urology, Sun Yat-Sen Memorial Hospital, Sun Yat-Sen University, Guangzhou, 510120 China; 2 Guangdong Provincial Key Laboratory of Malignant Tumor Epigenetics and Gene Regulation, Sun Yat-Sen Memorial Hospital, Sun Yat-Sen University, Guangzhou, 510120 China; 3 Guangdong Provincial Clinical Research Center for Urological Diseases, Sun Yat-Sen Memorial Hospital, Sun Yat-Sen University, Guangzhou, 510120 China; 4 Department of Urology, The Sixth Affiliated Hospital of Guangzhou Medical University, Qingyuan People’s Hospital, Qingyuan, 511518 Guangdong China; 5 Department of Urology, Nanfang Hospital, Southern Medical University, Guangzhou, 511430 China; 6 Department of Radiation Oncology, Sun Yat-Sen Memorial Hospital, Sun Yat-Sen University, Guangzhou, 510120 China; 7 Department of Urology, Fujian Medical University Union Hospital, Fuzhou, China

**Keywords:** Single-cell analysis, Prostate cancer, Docetaxel resistance, IL-11, Autocrine signalling

## Abstract

**Background:**

Docetaxel resistance represents a significant obstacle in the treatment of prostate cancer. The intricate interplay between cytokine signalling pathways and transcriptional control mechanisms in cancer cells contributes to chemotherapeutic resistance, yet the underlying molecular determinants remain only partially understood. This study elucidated a novel resistance mechanism mediated by the autocrine interaction of interleukin-11 (IL-11) and its receptor interleukin-11 receptor subunit alpha(IL-11RA), culminating in activation of the JAK1/STAT4 signalling axis and subsequent transcriptional upregulation of the oncogene c-MYC.

**Methods:**

Single-cell secretion profiling of prostate cancer organoid was analyzed to determine cytokine production profiles associated with docetaxel resistance.Analysis of the expression pattern of downstream receptor IL-11RA and enrichment of signal pathway to clarify the potential autocrine mechanism of IL-11.Next, chromatin immunoprecipitation coupled with high-throughput sequencing (ChIP-seq) was performed to detect the nuclear localization and DNA-binding patterns of phosphorylated STAT4 (pSTAT4). Coimmunoprecipitation and reporter assays were utilized to assess interaction between pSTAT4 and the cotranscription factor CREB-binding protein (CBP) as well as their role in c-MYC transcriptional activity.

**Results:**

Autocrine secretion of IL-11 was markedly increased in docetaxel-resistant prostate cancer cells. IL-11 stimulation resulted in robust activation of JAK1/STAT4 signalling. Upon activation, pSTAT4 translocated to the nucleus and associated with CBP at the c-MYC promoter region, amplifying its transcriptional activity. Inhibition of the IL-11/IL-11RA interaction or disruption of the JAK1/STAT4 pathway significantly reduced pSTAT4 nuclear entry and its binding to CBP, leading to downregulation of c-MYC expression and restoration of docetaxel sensitivity.

**Conclusion:**

Our findings identify an autocrine loop of IL-11/IL-11RA that confers docetaxel resistance through the JAK1/STAT4 pathway. The pSTAT4-CBP interaction serves as a critical enhancer of c-MYC transcriptional activity in prostate cancer cells. Targeting this signalling axis presents a potential therapeutic strategy to overcome docetaxel resistance in advanced prostate cancer.

**Graphical Abstract:**

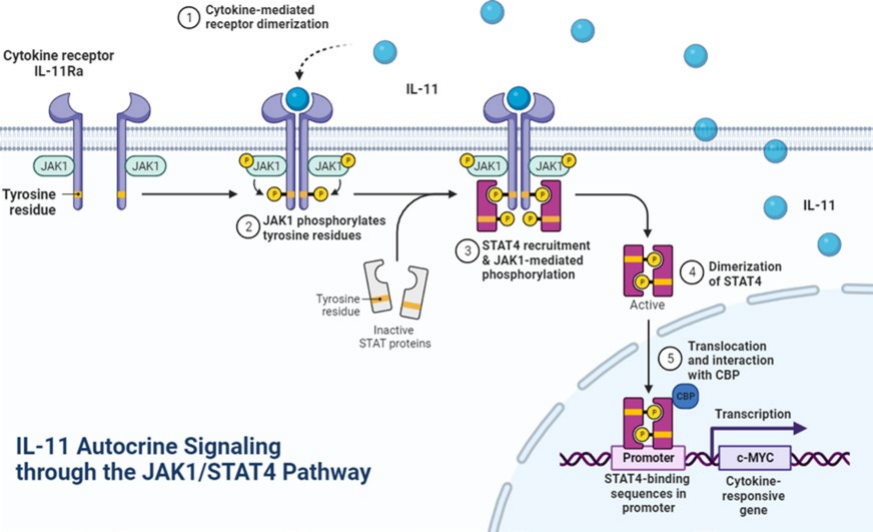

**Supplementary Information:**

The online version contains supplementary material available at 10.1186/s13046-024-02962-8.

## Introduction

Prostate cancer represents one of the most pressing health concerns in oncology, accounting for a significant proportion of cancer-related morbidity and mortality among men globally [1].The advent of docetaxel-based chemotherapy has marked a paradigm shift in the therapeutic landscape, offering a survival advantage in metastatic castration-resistant prostate cancer [2–4].Nonetheless, emergence of docetaxel resistance has inexorably become a predominant clinical hurdle, leading to treatment failure and cancer progression [5, 6].Circumvention of docetaxel resistance is thus imperative for improving patient outcomes, yet the molecular mechanisms driving this phenomenon remain incompletely understood.

In the realm of cancer biology, it is increasingly acknowledged that autocrine and paracrine signalling mediated by cytokines and growth factors is instrumental in sculpting tumour behaviour, influencing cellular proliferation, metastatic propensity, and sensitivity to chemotherapeutics [7–9]. Among the plethora of signalling molecules, interleukin-11 (IL-11), a cytokine in the IL-6 family, has gained attention due to its pernicious role in oncogenesis, promoting tumour cell proliferation, invasiveness, and drug resistance across diverse cancer types [10–12].The IL-11/IL-11RA interaction serves as the linchpin for this signalling cascade, typically culminating in activation of the Janus kinase/signal transducer and activator of transcription (JAK/STAT) pathway [13, 14], an axis known for its pivotal role in cellular survival and proliferation.

The JAK/STAT pathway, in particular, has been implicated in modulating cellular resistance to chemotherapeutic agents. Although STAT4's role in immunity is well characterized, its involvement in cancer pathophysiology, especially in orchestrating chemoresistance [13, 14], remains an area that needs to be investigated. At the heart of chemoresistance is intricate regulation of oncogenic transcription factors, among which c-MYC is crucial [15, 16].c-MYC, a master regulator of diverse cellular processes, including proliferation, metabolism, and apoptosis, is frequently found to be aberrantly overexpressed in prostate cancer and is closely associated with an unfavourable therapeutic outcome and resistance to conventional chemotherapy [17, 18].The transcriptional ability of c-MYC is governed not only by its expression but also by its interaction with cotranscription factors that modulate its transcriptional machinery.

Intrigued by the nexus between cytokine signalling and transcriptional control, we postulate that the IL-11 autocrine loop is a determinant of docetaxel resistance in prostate cancer via the JAK1/STAT4 signalling axis. This investigation delineates the molecular choreography of this resistance mechanism and sheds light on potential targets for therapeutic intervention, aiming to restore docetaxel sensitivity in prostate cancer cells resistant to this front-line chemotherapeutic agent.

## Methods

### Cell cultures

The human prostate epithelial cell line RWPE-1, the embryonic kidney cell line HEK-293 T, and the prostate cancer cell lines PC-3, LNCAP,22RV1,IE8 and DU145 were all acquired from American Type Culture Collection (ATCC, Manassas, Virginia, USA). The culture conditions for all cell lines adhered to previously established protocols [19, 20]. Rigorous quality control was maintained, with all cell lines testing negative for mycoplasma contamination. Additionally, to ensure the authenticity and purity of the cell lines, short tandem repeat (STR) authentication was performed, confirming no misidentification or contamination (verified by IGE Biotechnology, Guangzhou, Guangdong, China).For in vitro studies, PC3 or DU145 PCa cells, were stimulated with recombinant human IL-11 (Abcam,ab89887) or recombinant human IL-11RΑ (rhIL-11RΑ 10 ng/ml;Abcam,ab132575) for the indicated group, replacing culture medium and stimulus every 24 h.

### Immunohistochemistry (IHC) analyses

Immunohistochemistry (IHC) procedures were carried out according to established methodologies [21].In brief, tissue specimens underwent dewaxing and rehydration, followed by protease K incubation at 37 °C for 15 min to enable antigen retrieval. Subsequently, a 3% H2O2 solution was applied to the specimens for 10 min at 25 °C to inhibit endogenous peroxidase activity. The specimens were then exposed to primary antibodies overnight 4 °C. Detailed information regarding the primary antibodies utilized to assess protein expression can be found in Supplementary Table S1. After three washes with PBS, biotinylated secondary antibodies were applied to the specimens for an hour at 25 °C. DAB solution (sourced from ZSGB-BIO, Beijing, China) was then added for chromogenic detection. After colouration, haematoxylin was used for counterstaining.

Procedures from an earlier study were used for assessment of IHC [22]. Two pathologists, blinded to the specimen origins, quantified IL-11 expression by employing a designated staining scoring system. Specifically, the percentage of cancer cells displaying positive staining was determined. Subsequent immunostaining intensity within each specimen was categorized as follows: negative (score of 0), weak (score of 1), moderate (score of 2), or strong (score of 3). The resulting H-score was computed as the product of intensity and the percentage of positively stained cells. The specimens were then stratified based on IL-11 expression into low (score < 150) and high (score ≥ 150) groups. A similar approach was employed for evaluating JAK, STAT4, and Ki67 expression. Imaging was facilitated using the Nikon ECLIPSE Ti microscope system (Tokyo, Japan), and captured images were subsequently processed with proprietary Nikon software.

### RNA isolation, qPCR, and Western blotting

RNA isolation, quantitative real-time polymerase chain reaction (qPCR), and western blotting were performed in alignment with the methodologies in prior research [23, 24].For RNA processing, total RNA was extracted utilizing TRIzol Reagent (Takara, Kusatsu, Shiga, Japan), and 1 μg of RNA was reverse transcribed to yield complementary DNA (cDNA) employing the PrimerScript RT‒PCR kit (Takara). The ensuing qPCR assays involved use of SYBR Green reaction mix (Vazyme, Nanjing, Jiangsu, China) a LightCycler 96 System (Roche, Basel, Switzerland). The 2^^−ΔΔCt^ method was used for expression quantification, where Ct denotes the cycle threshold. The sequences for the specific primers used are provided in Supplementary Table S3.

For western blotting, cells were lysed in RIPA lysis buffer (CWBIO, Beijing, China) supplemented with protease and phosphatase inhibitors (CWBIO).Protein quantification was performed with Pierce BCA Protein Assay Kit (Invitrogen). The quantified proteins were subjected to SDS‒PAGE electrophoresis, transferred onto PVDF membranes (Merck, Burlington, Massachusetts, USA), and blocked. These membranes were then exposed to primary antibodies overnight at 4 °C. Details of the primary antibodies utilized are provided in Supplementary Table S2. After primary incubation, the membranes were treated with HRP-conjugated secondary antibodies at 25℃ for an hour. Finally, enhanced chemiluminescence was used for protein band visualization.

### Plasmids and transfection

We designed specific short hairpin RNA (shRNA) sequences targeting IL-11, c-MYC, STAT4 and CBP and incorporated them into the pLKO.1-Puro vector. All vectors for these procedures were procured from IGE (Guangzhou, Guangdong, China). To ascertain the fidelity of our cloning, we subjected the vectors to bidirectional sequencing. The specific sequences fused or the shRNAs are detailed in Supplementary Table S1.

For transient transfection, plasmid DNA was complexed with X-tremeGENE (Invitrogen) and allowed to incubate at 25 °C for 20 min. After incubation, this complex was introduced into cells, which were then maintained for 24–48 h. For lentivirus packaging, HEK-293 T cells were transfected with psPAX2 (IGE) and PMD2. G (IGE), and either stably silenced or overexpressed vectors using X-tremeGENE as the transfection reagent. After a 48-h interval, the lentiviruses were collected, subjected to filtration, and subsequently concentrated. Target cells were then exposed to these lentiviral particles in the presence of polybrene (IGE) and then selected using puromycin.

### Cell proliferation assays

To assess cell viability, we employed both MTT (3-(4,5-dimethylthiazol-2-yl)-2,5-diphenyltetrazolium bromide) and colony formation assays. Concurrently, we utilized EdU (ethynyl deoxyuridine) assays, leveraging the propensity of EdU to incorporate into DNA in place of thymidine during DNA replication in the S phase of the cell cycle and allowing us to quantify cells in the S phase. The methodologies for these assays refer to protocols we previously reported. In brief, a total of 1000 PCa cells from each group or transfected using siRNAs was added to 6‐well plates. After culture for 10 days, the colonies were fixed, stained and counted [24]. For MTT assays, we seeded 2 × 10^3 cells from varying experimental groups into individual wells of a 96-well plate. Next, a 20 μL aliquot of MTT solution (sourced from Sigma‒Aldrich, St Louis, MO, USA) was added to each well, and the cells were incubated for 3 h. After this incubation, the MTT solution was carefully removed, and 150 μL of dimethyl sulfoxide (DMSO) was added to each well to solubilize the formazan crystals. The absorbance, indicative of cellular viability, was then recorded at 490 nm using a spectrophotometer.

For colony formation assays, we placed 1000 prostate cancer (PCa) cells, either from different experimental groups or postsiRNA transfection, into each well of 6-well plates. After a 10-day incubation period, colonies that had formed were subjected to fixation, staining, and subsequent enumeration.

EdU assays were executed with EdU Kit (Catalogue: C10310-1, RIOBIO, Guangzhou, Guangdong, China). In brief, after the aforementioned treatments, PCa cells were placed in 96-well plates, with each well containing 3000 cells. A 4-h incubation with EdU followed, after which the cells were fixed, stained with Apollo®567, and visualized under fluorescence microscopy for analysis.

### Nuclear-Cytoplasmic fractionation

For nuclear-cytoplasmic separation, cells were collected and fractionated using a Nuclear/Cytosol Fractionation Kit (FUDE, FD0199, Hangzhou, China) according to the manufacturer's protocol. Briefly, cells were trypsinized, washed with PBS, and resuspended in cytoplasmic extraction buffer A. After incubation on ice and centrifugation, the supernatant (cytoplasmic extract) was collected. The pellet was resuspended in nuclear extraction buffer, and the nuclear extract was isolated following a series of incubation and centrifugation steps. The protein concentration was determined by the BCA assay (Abbkine, KTD3001, Wuhan, China).

### Immunoprecipitated DNA purification and qPCR analysis

Chromatin immunoprecipitation (ChIP) was performed in accordance with methodologies described [19].In brief, transfected cells were cross-linked using 1% formaldehyde for a duration of 10 min, followed by lysis employing SDS lysis buffer. Chromatin was subsequently sheared through ultrasonication, and aliquots of the supernatant were subjected to overnight immunoprecipitation at 4 °C using an antibody specific to phosphorylated STAT4. For the negative control, normal IgG was utilized (refer to Supplementary Table S2 for details). The Protein A/G beads complexed with the antibody and chromatin were then sequentially washed with buffers of varying ionic strengths, specifically low-salt, high-salt, and LiCl wash buffers, and finally with elution buffer to release the associated chromatin fragments. Cross-links within the DNA‒protein complexes were reversed, followed by DNA purification using spin-column chromatography. Enrichment of the target DNA sequences was quantified by quantitative PCR (qPCR). The sequences of the primers utilized for ChIP‒qPCR are detailed in Supplementary Table S3.

### Live/dead cell double staining assay analysis

Cell viability was assessed using a live-dead cell staining protocol. Cells were initially transfected with either an IL-11 overexpression plasmid or a control vector for 48 h, followed by treatment with docetaxel (DTX) for an additional 48 h. Subsequently, the culture medium was discarded, and viable cells were stained using Abbkine Live-Dead Cell Staining Kit (KTA1001, China) according to the manufacturer's instructions. After staining, visualization was performed using a fluorescence microscope (BX-50, Olympus). The abundance of viable cells was semiquantitatively evaluated based on the fluorescence intensity.

### Immunofluorescence (IF) staining

We conducted the IF staining in accordance with the methods outlined in our previous study [25]. In brief, PCa cells were seeded onto confocal dishes and fixed using 4% paraformaldehyde, followed by pre-hybridization with 0.5% Triton X-100. Subsequently, cells were blocked and incubated overnight with primary antibodies at 4 °C. The specific primary antibodies for anti-IL-11, anti-IL-11RA, anti-STAT4, and anti-p-STAT4 utilized in this research are detailed in Supplementary Table S2. Post-incubation, the dishes underwent a thrice-repeated washing with PBS, followed by a 1-h incubation with secondary antibodies at 25 °C. Cells were then stained with DAPI (Beyotime, Shanghai, China) for 5 min at 25 °C for nuclear counterstaining. Imaging was performed using a confocal microscope (Zeiss, Munich, Germany).

### ELISA‐based quantification of secreted IL-11

To measure levels of IL-11 secreted into the cell culture medium, we employed a Human ELISA Kit (Catalogue: ab100551, Abcam, Cambridge, UK) following the manufacturer's specific protocol [26]. In summary, cell culture supernatants from PCa cells were harvested and diluted in a 1:2 ratio. These diluted samples were then added to wells precoated with an IL-11-specific antibody. After a 30-min incubation at 37 °C, we recorded the absorbance for each well at 450 nm. We deduced the IL-11 concentration present in each well based on a standard curve.

### Gene ontology and Kyoto Encyclopedia of Genes and Genomes analysis

Gene Ontology (GO) and Kyoto Encyclopedia of Genes and Genomes (KEGG) are powerful tools used to infer potential biological processes and molecular pathways in which specific genes may participate. These analytical frameworks offer insights into the functional relevance of gene sets by categorizing them based on their involvement in biological process, cellular component, and molecular function terms (for GO) or by placing them within specific metabolic and signalling pathways (for KEGG). For the present study, both GO and KEGG analyses were executed using the online platform at https://www.bioinformatics.com.cn. This platform not only provides robust data analysis capabilities but also aids in visualization of the results, making it easy to discern patterns and relationships among the analysed genes. Data were accessed and processed on this platform on 10 July 2023.

### Prediction of response to chemotherapy

Predicting how individual tumour samples respond to chemotherapy and targeted drug treatments is a crucial aspect of precision oncology. To this end, we used the extensive Genomics of Drug Sensitivity in Cancer (GDSC2) database, one of the most comprehensive pharmacogenomics databases available, which can be accessed at https://www.cancerrxgene.org/. This database provides a vast reservoir of data, linking genomic profiles of cancer cell lines to their respective drug sensitivities.For our analysis, we utilized the "oncoPredict" package in R, a specialized tool designed for predicting drug sensitivities in oncological contexts. This package facilitates rigorous, methodical assessment of potential drug responses based on genomic data. Through regression analysis, we estimated the half-maximal inhibitory concentration (IC50) for each drug under consideration. The IC50 value serves as a benchmark, denoting the concentration of a drug needed to inhibit the growth of cancer cells by 50%. This provides a quantitative measure of the drug's potency and allowed us to gauge the relative effectiveness of different therapeutic agents against individual tumour samples.

### Unsupervised consensus clustering

To decipher distinct expression patterns among 15 prognostic differentially expressed genes (DEGs), we employed the R package ConsensusClusterPlus. This package is particularly designed to offer unsupervised consensus clustering, which serves as a technique to identify reproducible cluster structures in datasets, especially when dealing with high-dimensional genomic data. The consensus clustering methodology operates by repetitively sampling subsets of data (in our case, microarray data related to DEGs) and clustering these subsamples for a predefined number of clusters (denoted as 'k'). Once each subsampling and clustering iteration is completed, the algorithm computes pairwise consensus values. These values represent the proportion of times two specific items (genes in our context) are clustered together relative to the number of times they appear within the same sampled subset. All these consensus values are then collated into a symmetrical consensus matrix for each specified 'k'. This matrix serves as a comprehensive record of the clustering tendencies observed during the sampling iterations. To facilitate interpretation, the ConsensusClusterPlus package generates various graphical displays derived from the consensus matrix. These visual aids, including consensus heatmaps and cumulative distribution function (CDF) plots, are instrumental in assisting researchers in determining the optimal number of clusters for their dataset and understanding the cluster membership nuances.

### Application of gene set enrichment analysis

To delve deeper into the underlying biological implications of the identified differentially expressed genes (DEGs) between the IL11-high and IL11-low groups, we employed gene set enrichment analysis (GSEA). GSEA is a sophisticated computational method designed to determine if a priori defined sets of genes exhibit statistically significant, concordant differences between two biological states.

Robust nonparametric ensemble learning method tailored for survival analysis. For this task, we employed the 'Random Forest SRC' package, an advanced tool optimized for survival, regression, and classification trees. At the heart of this analysis is the random survival forest algorithm, which functions by generating numerous decision trees during its training phase and subsequently outputs an averaged result. To ensure comprehensive and rigorous assessment, we set the algorithm to undergo Monte Carlo simulation consisting of 2000 iterations (nrep = 2000). Such extensive repetition serves to stabilize importance measures, yielding reliable rankings of gene significance. Upon completion of the analysis, genes that exhibited a relative importance exceeding 0.1 were considered to be 'key genes'. This threshold ensures that only genes with a pronounced impact on prognosis, as indicated by their ability to consistently influence survival outcomes across the ensemble of decision trees, are spotlighted for further investigation.

### Single-cell deconvolution analysis

To dissect the heterogeneity within the tumor microenvironment, we utilized single-cell deconvolution algorithms. This involved employing computational approaches like those described in [27], to separate the mixed populations and analyze the contribution of each cell type to the overall gene expression.

### Xenograft tumor models

The animal experiments were conducted in strict adherence to the ethical guidelines approved by our hospital's Committee. We utilized 4-week-old male BALB/c nude mice as experimental subjects. These mice were injected with 1 × 10^^6^ PC3 cells, stably transfected with either shCtrl or shIL-11.Following the development of palpable tumors, the mice were randomly assigned into two groups (each *n* = 6) and received intraperitoneal injections of docetaxel at a dose of 10 mg/kg, administered twice weekly for a duration of three weeks.Tumor dimensions were measured bi-daily using calipers, and tumor volumes were calculated using the formula: V = 0.5 × length × width^^2^. Upon the completion of the study, the mice were humanely euthanized, and tumor tissues were harvested for further analysis.

### Data and code available

The transcriptomic profile pertaining to the docetaxel-resistant organoid models was sourced from the GEO (Gene Expression Omnibus) database, specifically the GSE162285,GSE137829,GSE141445 and GSE176031dataset.Additionally, we acquired the transcriptome profile and pertinent clinical information related to TCGA-PRAD from The Cancer Genome Atlas (TCGA), which can be accessed at https://portal.gdc.cancer.gov/. This particular dataset was retrieved on 29th October 2022. In the spirit of fostering scientific transparency and collaboration, we wish to note that all computational codes employed throughout our study are readily available. Researchers and interested parties can obtain these by placing a reasonable request directed to the corresponding author of this article.

### Statistical analysis

For analysis of categorical variables, we utilized either the chi-square test or Fisher’s exact test, depending on the data distribution and sample size. Continuous variables were analysed using the t test or the Wilcoxon rank-sum test, contingent on whether the data met the assumptions of normality. Survival outcomes, including recurrence-free survival (RFS) and overall survival (OS), were assessed using Kaplan‒Meier analysis. This nonparametric statistic offers a visual representation of the survival data and allows for comparisons between groups to discern significant differences in survival probabilities. All statistical procedures were conducted using R software, version 4.2.1, which is a comprehensive suite for statistical computing and graphics. It was developed and maintained by The R Foundation for Statistical Computing and can be accessed at http://www.r-project.org/.

In our analysis, we adhered to conventional significance thresholds: a two-sided *P* value below 0.05 indicated statistical significance for all assessed outcomes.

## Results

### Differential Gene Expression in Docetaxel-Resistant Prostate Cancer Organoid Models

**Fig. 1:**
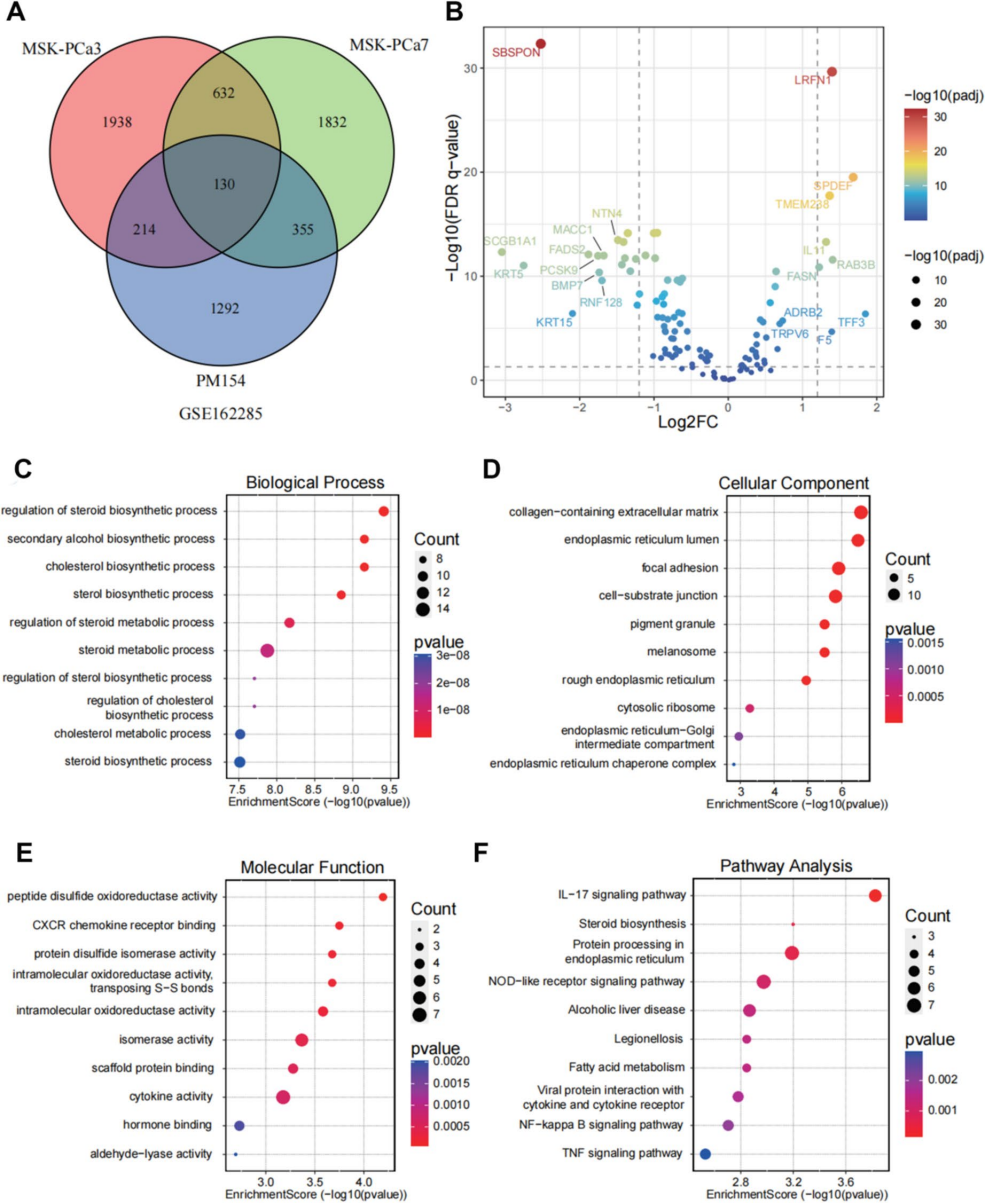
Transcriptomic profiling and functional enrichment analysis in docetaxel-resistant prostate cancer organoid models. **A** Venn diagram illustrating the overlap of differentially expressed genes (DEGs) in MSK-PCa3, MSK-PCa7, and PM154 prostate cancer cell lines, as referenced from the GSE162285 dataset. **B** Volcano plot displaying DEGs in MSK-PCa cell lines. The x-axis represents the log2-fold change (log2FC), while the y-axis shows the negative log10 of the false discovery rate (FDR)-adjusted *p* value (-log10(FDR *p* value)). Genes with significant differential expression are highlighted; those with Log2FC > 1 and -log10(FDR *p* value) > 1.5 are in red (upregulated), while those with Log2FC < -1 and -log10(FDR *p* value) > 1.5 are in green (downregulated). **C**, **D** Bubble chart of enriched biological processes and cellular components among DEGs, with bubble size indicating gene count and colour representing *p* value significance. **E**, **F** Molecular function enrichment analysis and pathway analysis bubble chart of DEGs presented as a bubble chart, where bubble size indicates the gene count and colour the *p* value significance

In our quest to elucidate the mechanisms for docetaxel resistance in prostate cancer, we initiated our analysis by examining the RNA expression profiles of three prostate cancer organoids—namely, MSK-PCa3, MSK-PCa7, and PM154. These expression profiles were extracted from the GSE162285 dataset. Employing the limma package in R, we conducted differential expression analysis between docetaxel-resistant samples and their treatment-naive counterparts across the three organoid models. We instituted stringent criteria for significance, setting the threshold for differential expression at a *P* value of less than 0.05. Through this rigorous analytic approach, we identified a set of 130 genes that were consistently differentially expressed across all three organoid models (Fig. 1 A).

To further contextualize our findings, we juxtaposed the RNA expression profiles of these 130 genes against their profiles in tumour and adjacent normal tissues from the TCGA-PRAD cohort. Interestingly, 117 of the 130 genes identified from the organoid models were detectable in the TCGA-PRAD dataset, underscoring their potential relevance in a broader clinical context (Fig. 1, B). To decode the potential functional implications of these differentially expressed genes (DEGs), we subjected them to Gene Ontology (GO) and Kyoto Encyclopedia of Genes and Genomes (KEGG) pathway analyses. Our findings revealed notable enrichment of these genes in lipid metabolism pathways, processes associated with cellular organelles, and pathways pertinent to cytokine signalling and immune system modulation (Fig. 1, C-F). Collectively, our analyses shed light on a unique gene signature that might play a pivotal role in development of docetaxel resistance in prostate cancer organoid models, offering avenues for future therapeutic interventions.

### Vital DEGs associated with prognosis of prostate cancer

**Fig. 2:**
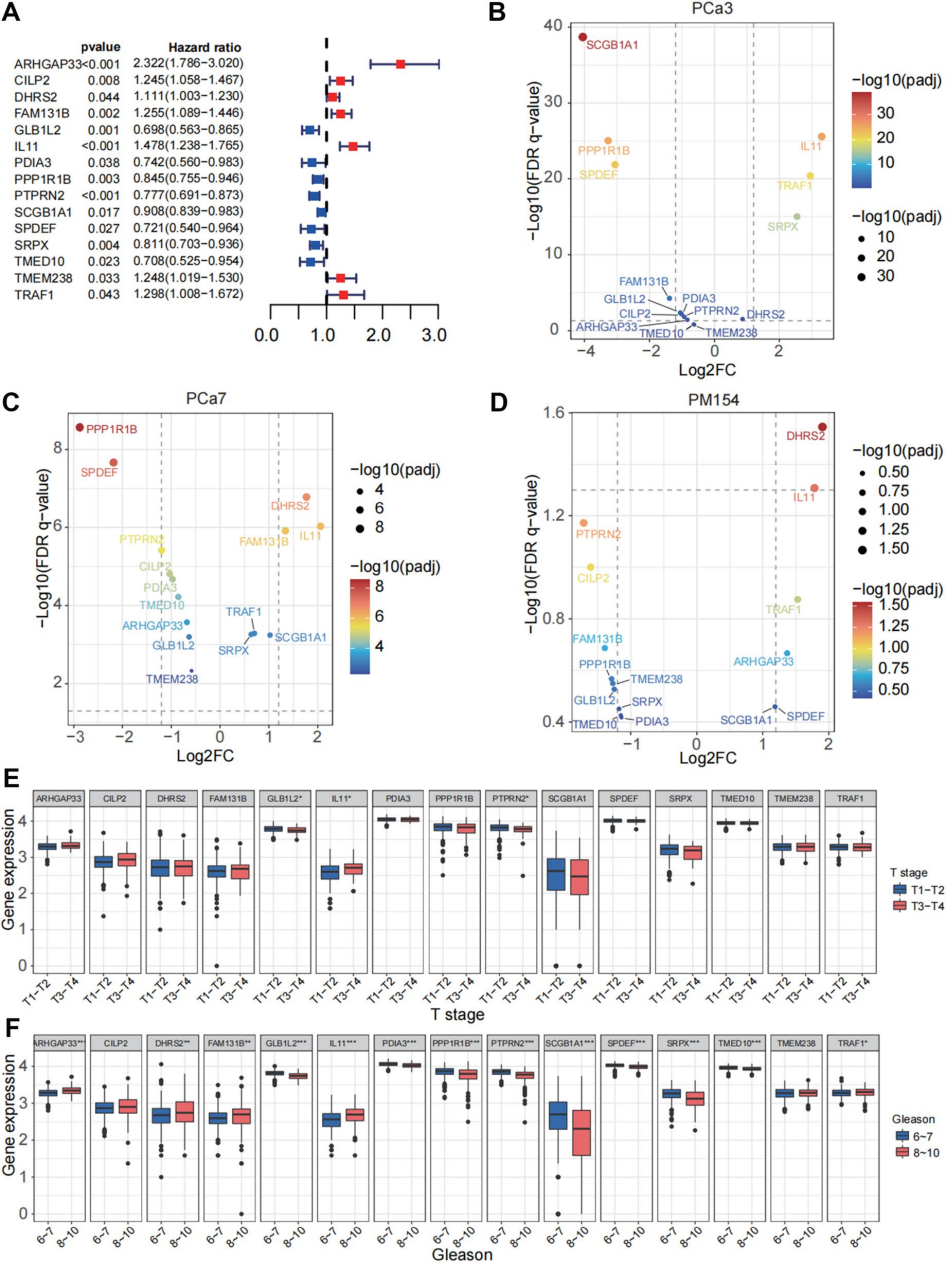
Prognostic and expression analysis of candidate genes in prostate cancer. **A** Forest plot representing the hazard ratios and *p* values of candidate genes associated with overall survival in prostate cancer. The horizontal lines correspond to the 95% confidence intervals, and the squares indicate the hazard ratio (red for increased risk; blue for decreased risk). Genes with a hazard ratio greater than 1 suggest a higher risk, while those with a hazard ratio less than 1 suggest a protective effect. **B**-**D** Volcano plots illustrating differentially expressed genes (DEGs) in docetaxel-resistant prostate cancer cells: PCA3 (**B**), PCA7 (**C**), and PM154 (**D**). The x-axis shows the log2-fold change (log2FC), while the y-axis represents the negative log10 of the false discovery rate (FDR)-adjusted p value (-log10(FDR p value)). The colour gradient represents the significance level of the p value, with warmer colours indicating higher significance. **E** Box plots depicting the expression levels of the candidate genes across different T stages (T1-T2 vs. T3-T4) of prostate cancer. Each box plot shows the median, quartiles, and range of expression values. **F** Box plots showing expression levels of the candidate genes across different Gleason scores (6–7, indicating less aggressive tumours, vs. 8–10, indicating more aggressive tumours) in prostate cancer. Each box plot represents the median, quartiles, and range of expression values. In all panels, *p < 0.05, **p < 0.01, ***p < 0.001 indicate statistical significance. The size of the dots in volcano plots (B-D) corresponds to the level of significance, with larger dots representing more significant p values. Gene expression data were normalized, and statistical tests were adjusted for multiple comparisons using the Benjamini‒Hochberg method

After obtaining the transcript profile of 117 DEGs associated with docetaxel resistance in the TCGA-PRAD cohort, we next identified vital genes related to the prognosis of patients. Through univariate Cox regression analysis, 15 genes were significantly associated with biochemical recurrence (BCR) of prostate cancer, among which ARHGAP33, CILP2, DHRS2, FAM131B, IL11, TMEM238, and TRAF1 were identified as risk factors (with hazard ratio > 1) and GLB1L2, PDIA3, PPP1R1B, PTPRN2, SCGB1A1, SPDEF, SRPX, and TMED10 as protective factors (with hazard ratio > 1) (Fig. 2 A, S2C). Figure 2 B-D displays differential expression of these 15 genes in the docetaxel-resistant group compared to the nonresistant group in the three organoid models. Among risk factor genes, IL11, TRAF1, and DHRS2 were upregulated in the docetaxel-resistant group, indicating consistency with progression of tumour docetaxel resistance and poor prognosis. Among protective genes, GLB1L2, PDIA3, PPP1R1B, PTPRN2, and TMED10 were downregulated in the docetaxel-resistant group. In the TCGA-PRAD cohort, expression of IL11 was significantly increased in patients with higher T stage but that of GLB1L2 and PTPRN2 significantly reduced (Fig. 2 E). ARHGAP33, DHRS2, FAM131B, IL11, and TRAF1 were significantly increased in patients with high Gleason scores, while GLB1L2, PDIA3, PPP1R1B, PTPRN2, SCGB1A1, SPDEF, SRPX, and TMED10 were significantly reduced in patients with high Gleason scores (Fig. 2 F).

### IL-11 is upregulated in PCA tissues and cell lines and related to docetaxel resistance and biochemical recurrence

**Fig. 3:**
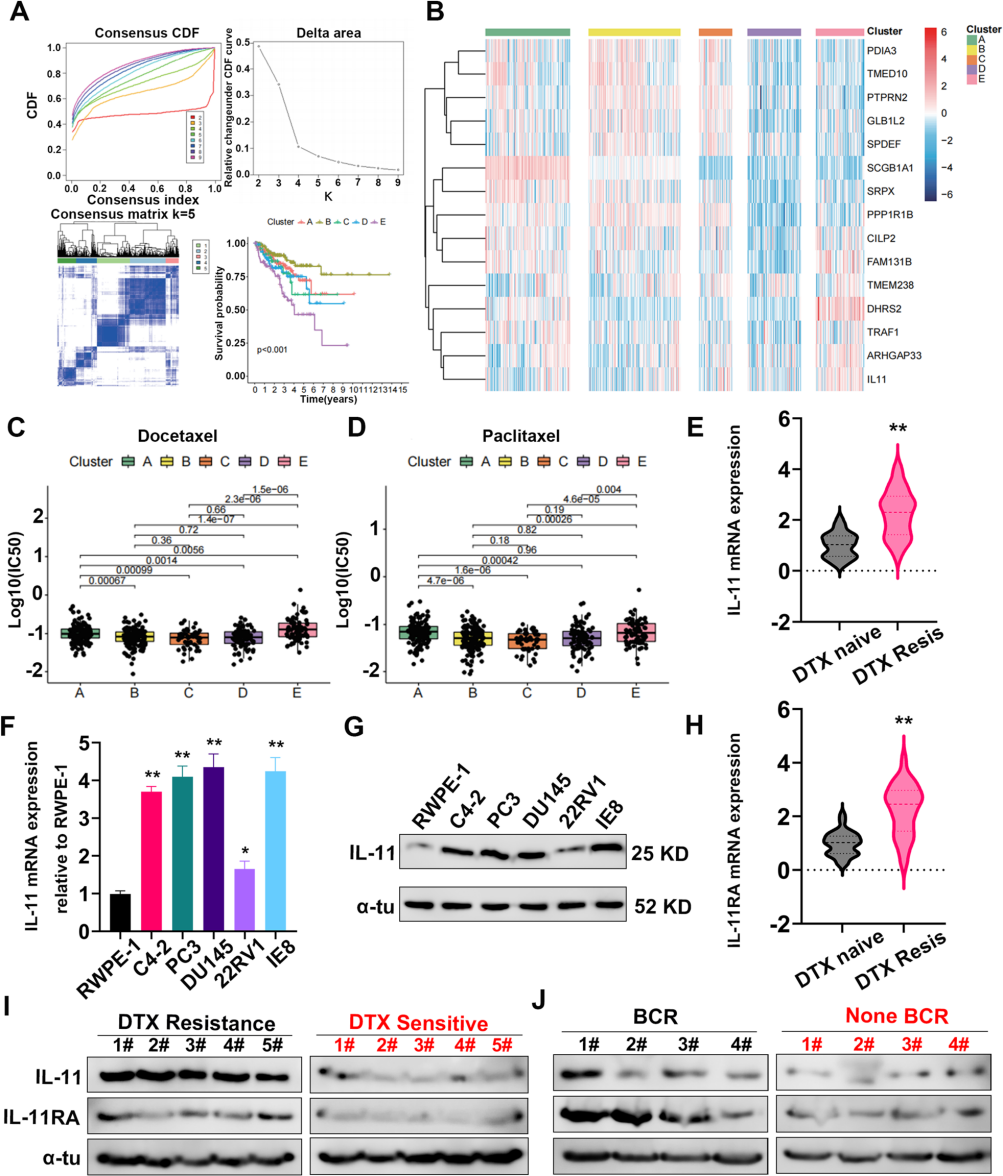
IL-11 is upregulated in prostate cancer and associated with docetaxel resistance and biochemical recurrence. **A** Top panel: Kaplan‒Meier survival curves for prostate cancer patients stratified into subtypes A-E based on gene expression profiles, with the log-rank *p* value indicating significant differences in survival outcomes. Middle panel: A heatmap of the top differentially expressed genes (DEGs) across the identified subtypes, with hierarchical clustering on the y-axis and individual genes on the x-axis. Bottom panel: Box plots illustrating differential drug sensitivity among the subtypes for various chemotherapeutic agents, as measured by the log-transformed half-maximal inhibitory concentration (log(IC50)). **B** A comprehensive heatmap representing normalized gene expression values of key DEGs across prostate cancer subtypes A-E, with the dendrogram indicating the clustering of subtypes based on gene expression patterns. **C**, **D** Box plots comparing the sensitivity of prostate cancer subtypes to different chemotherapeutic agents, including docetaxel and paclitaxel. The y-axis represents the log(IC50) values indicating drug sensitivity, while the x-axis denotes the subtypes. **E** Violin plot depicting the elevated mRNA expression of IL-11 in docetaxel-resistant (DTX Resis) compared to naïve prostate cancer samples.(*n* = 56) (**F**) IL-11 mRNA and protein expression (**G**) were detected by RT–PCR and western blot analysis in the RWPE-1, C4-2, PC3, DU145, 22RV1 and IE8 cell lines. **H** Box plot illustrating the increased mRNA expression of IL-11RA in docetaxel-resistant vs. naïve samples (*n* = 56) (**I**) The protein level of IL-11/IL-RA from PCA patients with (n = 5) or without (*n* = 5) docetaxel resistance was examined by western blotting analysis. J Western blots comparing expression levels of IL-11 and its receptor IL-11RA from PCA patients categorized as either resistant or sensitive to docetaxel (DTX) in Part I and in the context of BCR (biochemical recurrence) status in Part J, with α-tubulin as a loading control. *P < 0.05, **P < 0.01, ***P < 0.001

To obtain different genotypes of docetaxel resistance, we conducted unsupervised consensus clustering analysis based on expression of the 15 genes in the TCGA-PRAD cohort, and patients were divided into five different clusters (Fig. 3 A-C). Cluster A featured high expression of SCGB1A1. Cluster B highly expressed protective factors such as PDIA3 and GLB1L2, with less expression of risk factors IL11, TRAF1, and DHRS2. Cluster C showed less expression of SCGB1A1, with moderate expression of other genes. Cluster D exhibited low expression of all 15 genes. Cluster E highly expressed risk factors such as IL11, TRAF1, and DHRS2, with less expression of protective factors such as PDIA3 and GLB1L2. Kaplan‒Meier curves indicated that the BCR-free survival of patients in Cluster E was shortest and that patients in Cluster B had the longest BCR-free survival. We used the R package “OncoPredict” to predict the sensitivity of each group to chemotherapy and targeted drugs and found that patients in Cluster E had the highest IC50 values for docetaxel and paclitaxel. These results were consistent with outcomes in organoid models of GSE162285, indicating that high expression of IL-11, ARHGAP3, TRAF1, DHRS2, FAM131B, and CILP2 correlated positively with docetaxel resistance. Moreover, the sensitivity of each group to drugs such as 5-fluorouracil and olaparib also varied, as described in the additional materials (Figure S3A-G).

We further assessed the expression level of IL-11 in PCA cell lines (C4-2, PC3, DU145, 22RV1 and IE8) and normal prostatic epithelial cells (RWPE-1). As shown in Fig. 3F, G, we observed a significant elevation in both mRNA and protein levels of IL-11 in PCA cells compared to the RWPE-1 cell line. Additionally, our analysis of clinical PCA samples revealed a marked overexpression of IL-11 and IL-11RA in prostate cancer tissues versus adjacent non-cancerous tissues. This overexpression was particularly pronounced in samples from prostate cancer patients resistant to DTX treatment, as depicted in Figs. 3E, H, and Supplementary Figure S3H, I. Consistently, there was a higher IL-11/IL-11RA protein level in the tissues of patients with PCA with docetaxel resistance/biochemical recurrence than in those of patients without docetaxel resistance/biochemical recurrence (Fig. 3I, J).

### IL-11 contributes to increased viability and chemoresistance in prostate cancer cells

**Fig. 4:**
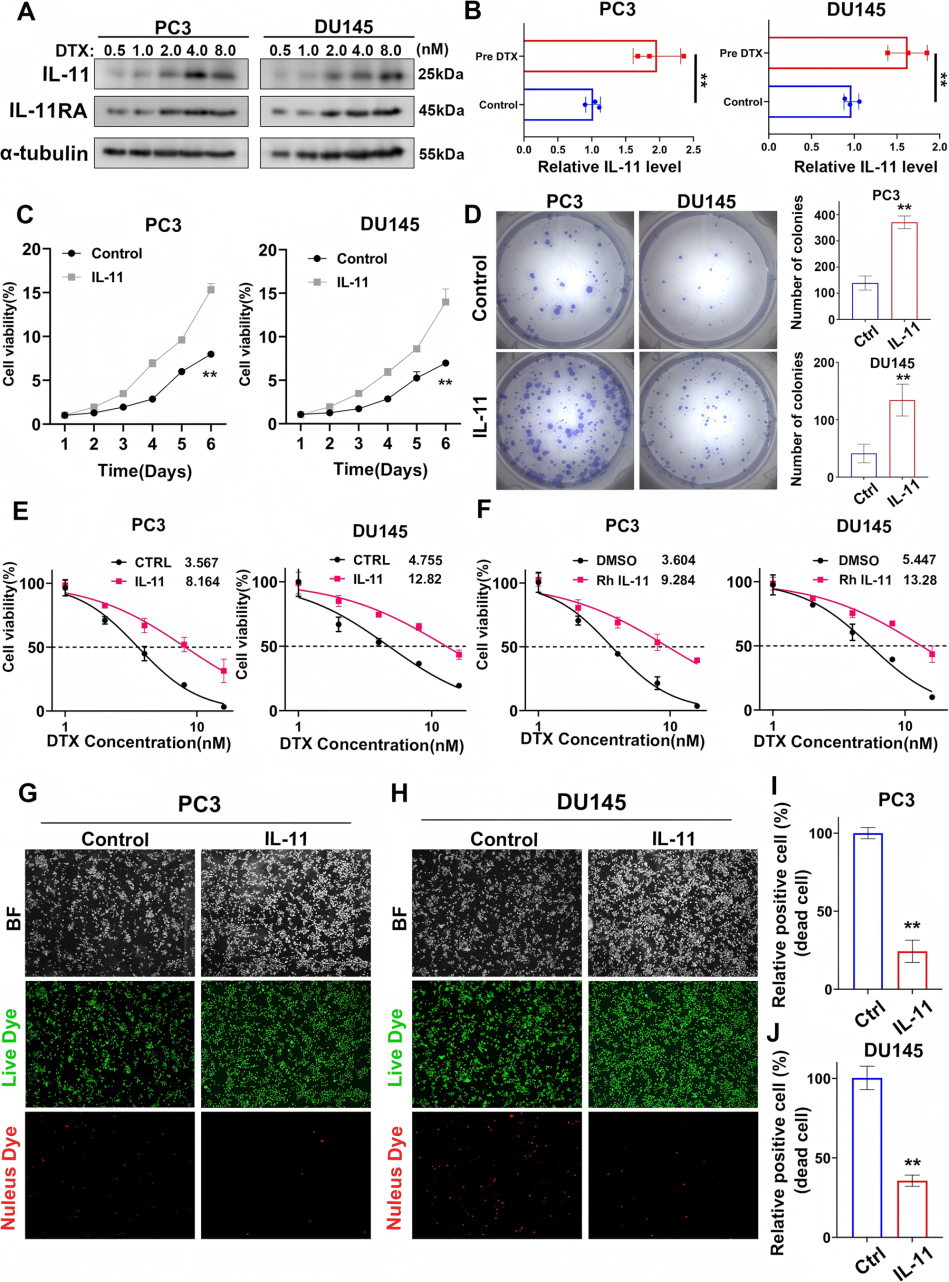
IL-11 contributes to increased viability and chemoresistance in prostate cancer cells. **A** Western blot analyses displaying the dose-dependent expression of IL-11 and IL-11RA in prostate cancer cell lines PC3 and DU145 after treatment with various concentrations of docetaxel (DTX) for 48 h. **B** ELISA measuring IL-11 secretion in the prostate cancer cell lines PC3 and DU145 with or without DTX treatment(1 nM for 48 h). The error bars indicate the standard deviations of three experiments independently. **C** Line graphs representing the percentage of cell viability over a period of 6 days for prostate cancer cell lines PC3 and DU145, comparing the effects of IL-11 overexpression against the control. **D** Images of colony formation in prostate cancer cell lines PC3 and DU145, comparing the control and overexpression treated groups, alongside bar graphs quantifying the number of colonies formed, indicating a significant increase in colony formation upon IL-11 treatment. **E** Graphs plotting cell viability percentages against a range of DTX concentrations for prostate cancer cell lines PC3 and DU145, comparing control groups to those treated with IL-11 overexpression. **F** Graphs depicting the effect of recombinant human interleukin 11(RhIL-11) on the cell viability of prostate cancer cell lines PC3 and DU145 at various DTX concentrations compared to the DMSO control. **G**, **H** Microscopy images of prostate cancer cell lines PC3 (**G**) and DU145 (**H**) displaying live (green) and dead (red) cells in the control and IL-11-treated groups, as indicated by bright field and fluorescence staining (**I**, **J**). Bar graphs showing the percentage of dead cells in prostate cancer cell lines PC3 (I) and DU145 (**J**), comparing the control and IL-11-treated groups. *P < 0.05, **P < 0.01, ***P < 0.001

To ascertain the impact of interleukin-11 (IL-11) on the phenotypic attributes of prostate cancer cell lines, we conducted a series of experiments on PC3 and DU145 cells. Western blot analysis revealed a dose-dependent increase in IL-11 and IL-11RA proteins following docetaxel (DTX) administration, implicating IL-11 in development of DTX resistance (Fig. 4A). This was supported by ELISAs, which detected significant IL-11 secretion after DTX treatment (Fig. 4B).

Subsequent investigations utilizing cell viability and colony formation assays indicated a pronounced enhancement in cell survival and proliferation upon IL-11 overexpression, particularly under chemotherapy stress (Fig. 4C, D). Cells with IL-11 overexpression demonstrated superior viability over a period of six days and a substantial increase in colony formation relative to the untreated controls. Additionally, the data revealed that recombinant human IL-11 (Rh IL-11) exerted a cytoprotective effect against a range of DTX concentrations, indicating a potential mechanism by which IL-11 confers a survival benefit to prostate cancer cells (Fig. 4E, F). Live/dead assays further corroborated these observations, showing a marked decrease in mortality rates in IL-11-treated cells, thus emphasizing the role of IL-11 in promoting a chemoresistant phenotype (Fig. 4G-I).

### The autocrine loop of IL-11/IL-11RA in promoting cell survival and potential resistance mechanisms to Docetaxel

**Fig. 5:**
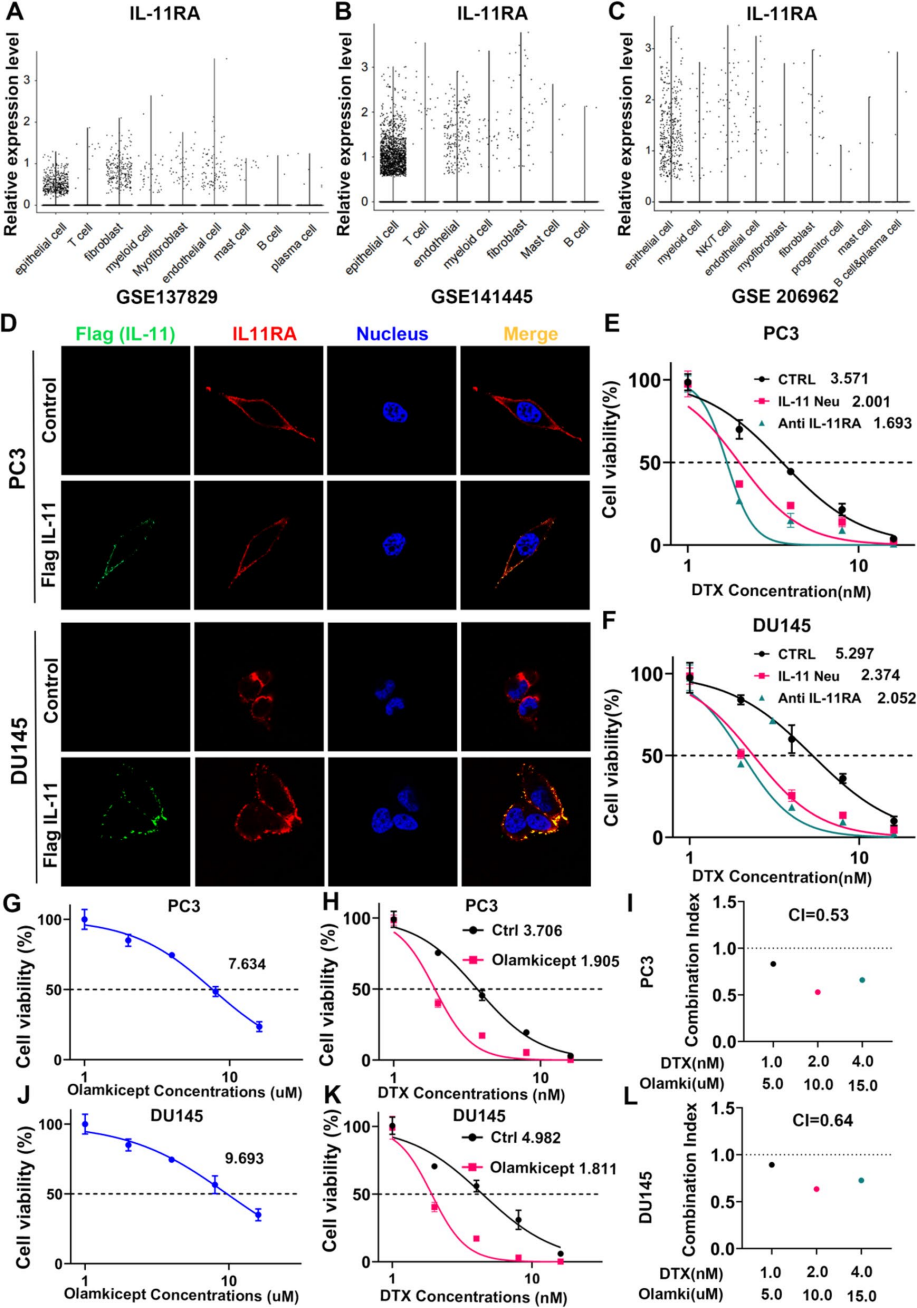
The autocrine loop of IL-11/IL-11RA in promoting cell survival and potential resistance mechanisms to docetaxel. (**A**-**C**) Dot plots representing relative expression levels of IL-11RA across various prostate cancer cell lines and clinical samples, sourced from datasets GSE137829, GSE141445, and GSE176031, respectively. Each dot represents an individual sample, and the spread indicates variability within the sample set. **D** Prostate cancer cells (PC3 and DU145) transfected with the control empty vector or Flag IL-11 overexpression plasmid were cocultured with wild-type prostate cancer cells for 48 h, and the images show the cellular localization of Flag-tagged IL-11 (green), IL-11RA (red), and cell nuclei (blue). Merged images illustrate the colocalization of IL-11 with its receptor. **E**, **F** Panels E (PC3 cells) and F (DU145 cells) plot cell viability percentages against docetaxel (DTX) concentrations. Comparisons were made between control cells treated with an IL-11 neutralization antibody (IL-11 Neu) or an IL-11 antagonist (anti-IL-11RA). **G**, **J** Panels G (PC3 cells) and J (DU145 cells) show the effect of various concentrations of olamkicept, an IL-11 signalling inhibitor, on cell viability. **H**, **K** Panels H (PC3 cells) and K (DU145 cells) illustrate the cell viability percentages in response to combined treatment with docetaxel and ouakicept, suggesting potential synergistic effects. **I**-**L** Panels I (PC3 cells) and L (DU145 cells) present the combination index (CI) for cotreatment with docetaxel and ouakicept at various concentrations, providing a quantitative measure of the drug interaction, where a CI less than 1 indicates synergy

Examination of single-cell transcriptome sequencing datasets GSE137829, GSE141445, and GSE176031 highlighted the expression level of IL-11/IL-11RA across diverse cells, suggesting an autocrine role for IL-11/IL-11RA signalling in prostate cancer cells and its influence on the therapeutic response to docetaxel (Fig. 5A-C, Figure S1A-I).

Furthermore, we visualized the interaction between IL-11 and its receptor IL-11RA within PC3 and DU145 cell lines modified to overexpress Flag-tagged IL-11 and cocultured them with wild-type cells. Immunofluorescence images revealed colocalization of the ligand‒receptor pair (Fig. 5D), providing insight into the spatial dynamics of IL-11 signalling within the tumour microenvironment. We further quantified cell viability in response to a gradient of docetaxel concentrations, noting a significant reduction in cell survival upon disruption of IL-11/IL-11RA signalling by either an IL-11 neutralizing antibody or an antagonist (Fig. 5E, F). These findings underscore the pivotal role of autocrine IL-11/IL-11RA signalling in mediating resistance to docetaxel, presenting a potential avenue for therapeutic intervention.

While exploring this therapeutic avenue, we assessed the impact of olamkicept, an inhibitor of IL-11 signalling, on cell viability (Fig. 5G, J). The results indicated a dose-dependent decrease in the viability of both PC3 and DU145 cells, which was further potentiated when olamkicept was used in combination with docetaxel (Fig. 5H, K). The synergy between these agents was quantitatively supported by combination index values below 1, suggesting a novel combinatorial approach to enhance the cytotoxic effects of docetaxel in prostate cancer therapy (Fig. 5I, L). This synergistic relationship not only reinforces the contributory role of IL-11/IL-11RA signalling in chemoresistance but also showcases the potential of targeted therapies to improve clinical outcomes in prostate cancer.

### IL-11 enhanced prostate cancer (PCa) progression and docetaxel resistance in vivo

**Fig. 6:**
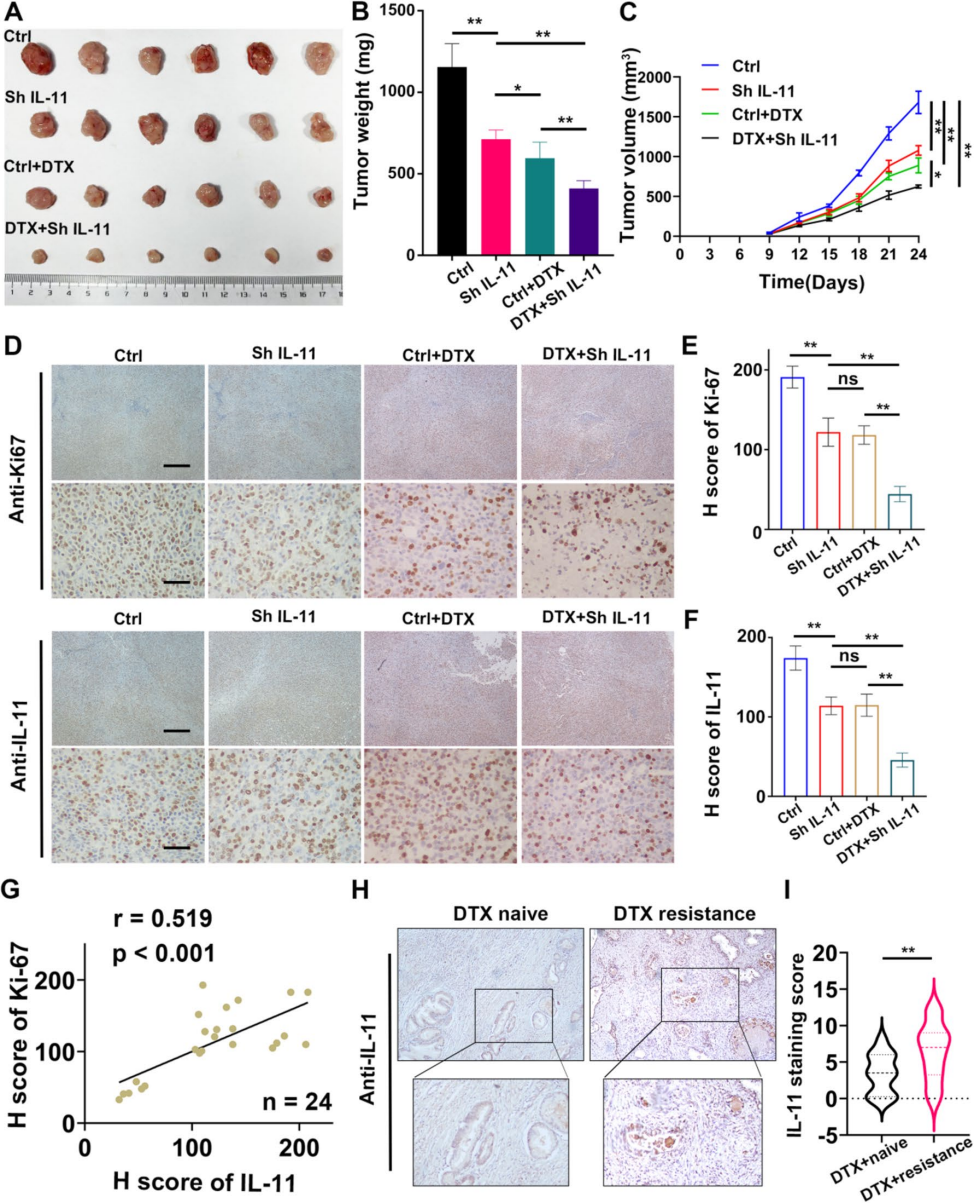
IL-11 enhanced prostate cancer (PCa) progression and docetaxel resistance in vivo. **A** Representative image of subcutaneous tumours in the control, IL-11 knockdown, docetaxel-treated (Ctrl + DTX), and dual-treated (DTX + Sh IL-11) groups. **B** Histogram showing tumour weights in the control, IL-11 knockdown, Ctrl + DTX and DTX + Sh IL-11 groups after surgical dissection. **C** Growth of the tumours in the control, IL-11 knockdown, Ctrl + DTX and DTX + Sh IL-11 groups was measured every 3 days, and tumour growth curves were calculated. The mean ± standard deviation (SD) of the tumour volumes measured in 6 mice is shown. **D** Immunohistochemical staining showing IL-11 and Ki67 expression in tumours. **E**, **F** Histogram showing the H‐scores of Ki-67 and IL-11 in the control, IL-11 knockdown, Ctrl + DTX and DTX + Sh IL-11 groups. The error bars represent the standard deviations of values in each group (*n* = 6). **G** Pearson correlation analysis between IL-11 and Ki67 expression. **H**, **I** Comparative immunohistochemical analysis of IL-11 expression in docetaxel-resistant clinical prostate cancer tissues versus naive counterparts, as shown in representative images and quantified in a violin plot (*n* = 16)

Subsequently, we introduced prostate cancer cells with stable IL-11 knockdown and cells with concurrent docetaxel treatment into a murine model. Abrogation of IL-11 signalling markedly impeded tumour progression in vivo, as evidenced by attenuated tumour growth in the IL-11-silenced cohort relative to the controls. Conversely, docetaxel treatment alone demonstrated some therapeutic efficacy, but the most pronounced antitumour effect was observed in the group subjected to the combination of IL-11 knockdown and docetaxel (Fig. 6A). Tumours derived from the dual-treated group not only grew at a slower pace but also weighed significantly less than those in the control and single-treatment groups (Fig. 6 B, C). Immunohistochemical analysis reflected this trend at the molecular level, with the lowest Ki67 and IL-11 expression detected in the dual-treated group, indicating dampened proliferative activity and signalling (Fig. 6 D-G). In the context of docetaxel resistance, IL-11 expression was heightened in resistant clinical specimens as opposed to naive specimens, a disparity that was illustrated and quantified through immunohistochemical assessments (Fig. 6H, I). In aggregate, these data highlight the pivotal role of IL-11 in facilitating prostate cancer cell proliferation and tumour growth and underscore the therapeutic potential of targeting IL-11 in conjunction with established chemotherapy regimens.

**Fig. 7:**
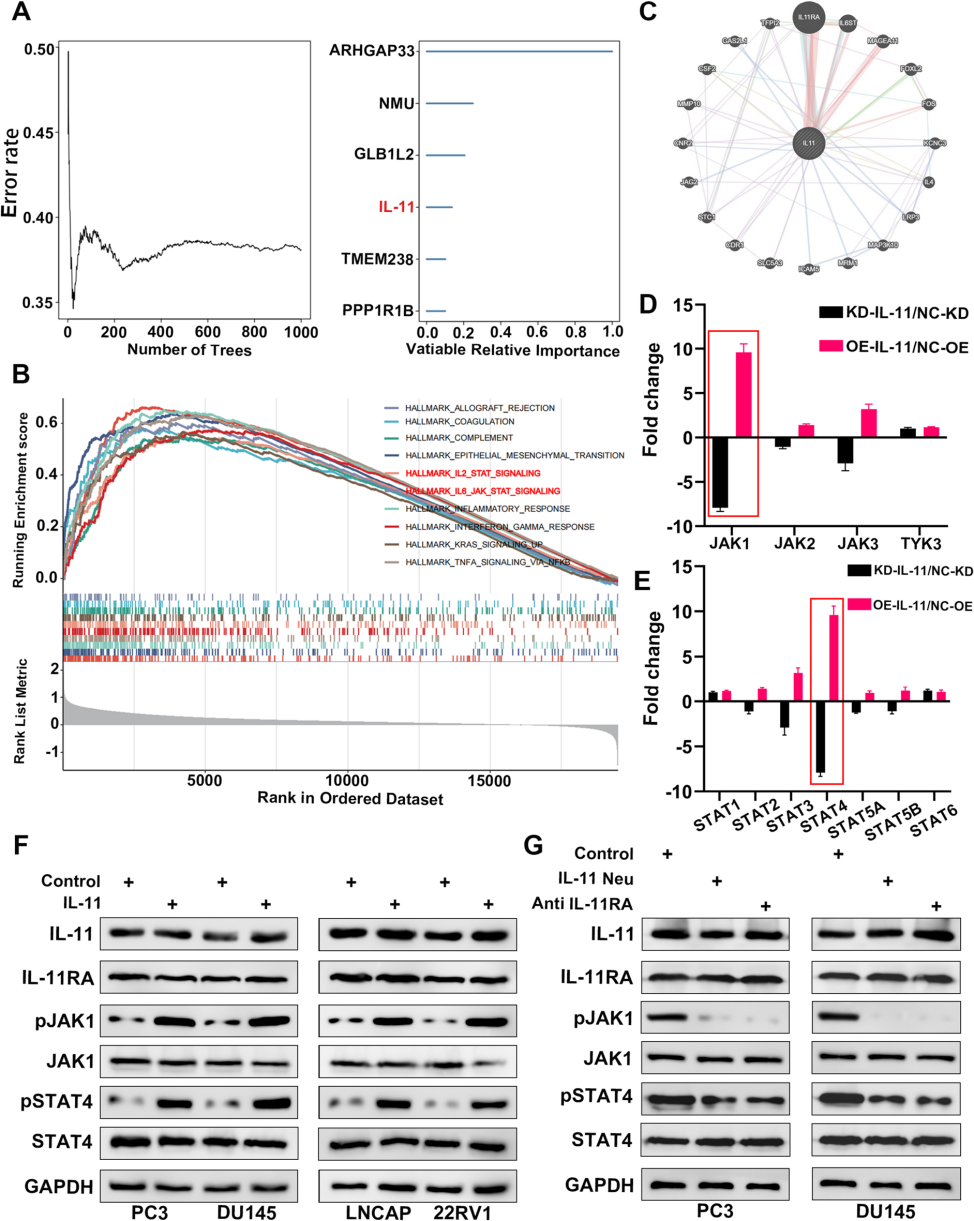
Analysis of IL-11-mediated signalling in prostate cancer cell lines. **A** Random forest analysis indicating the error rate as a function of the number of trees, with key genes labelled for their variable importance in the model. **B** Gene set enrichment analysis (GSEA) showing the running enrichment scores for selected hallmark gene sets across a ranked list of genes from the dataset. The JAK/STAT signalling pathway is emphasized, indicating its involvement in the response to IL-11. **C** Network representation of the protein‒protein interaction (PPI) focused on IL-11, with edge colours representing the type of interaction and node size correlating with the number of interactions per protein. **D** Bar graph illustrating the fold change in expression levels of JAK family kinases (JAK1, JAK2, JAK3, TYK3) upon alterations in IL-11 in prostate cancer cell lines. **E** Bar graph representing the fold change in expression levels of STAT family proteins (STAT1, STAT2, STAT3, STAT4, STAT5A, STAT5B, STAT6) upon alteration of IL-11 in the same cell lines. **F** Western blot analysis showing protein expression of IL-11 and IL-11RA and the phosphorylation status of JAK1 and STAT4 in response to IL-11 treatment across different prostate cancer cell lines (PC3, DU145, LNCAP, 22RV1). G: Western blot analysis comparing protein expression and phosphorylation of JAK1 and STAT4 upon neutralization of IL-11 or IL-11 antagonist in PC3 and DU145 cell lines

In our investigation into the role of IL-11 in prostate cancer signalling pathways, we employed a multifaceted analytical approach. By utilizing a random forest algorithm, we discerned a clear correlation between IL-11 signalling and the activation of key genes within the JAK/STAT pathway. Our model’s accuracy improved with the number of trees, highlighting the importance of IL-11 along with several other genes, such as ARHGAP33 and NMU, which were identified as significant variables in the signalling model (Fig. 7A). Gene set enrichment analysis (GSEA) further corroborated these findings by demonstrating substantial enrichment of the JAK/STAT signalling hallmark gene set following IL-11 stimulation. This enrichment was evident across a spectrum of genes ranked according to their expression profiles in prostate cancer cell lines, signifying the pathway's active role in response to IL-11 (Fig. 7B). Moreover, a protein‒protein interaction network explicitly centred around IL-11 revealed extensive interactions, with network density suggesting a prominent regulatory role for IL-11 in the signalling milieu of prostate cancer (Fig. 7C).

Alterations in IL-11 levels resulted in notable changes in the expression of JAK family kinases, with JAK1, JAK2, JAK3, and TYK3 all showing differential expression patterns. The fold changes observed were significant and suggested a significant modulation of STAT1 by IL-11 (Fig. 7D, S2A). Parallel changes were noted in STAT family proteins, with STAT4 in particular demonstrating a significant response to IL-11 modulation, thereby reinforcing the connection between IL-11 signalling and STAT4-mediated transcriptional regulation in prostate cancer (Fig. 7E, S2B). Western blot analyses across various prostate cancer cell lines, including PC3, DU145, LNCAP, and 22RV1, further demonstrated that IL-11 treatment modulates not only expression of IL-11 and its receptor IL-11RA but also the phosphorylation status of downstream signalling molecules such as JAK1 and STAT4, suggesting an autocrine active IL-11/IL-11RA signalling axis in these cells (Fig. 7F). Notably, upon neutralization of IL-11 signalling using an IL-11 antagonist, the expression and phosphorylation levels of JAK1 and STAT4 were markedly altered, substantiating the pivotal role of IL-11 in activation of the JAK/STAT pathway in prostate cancer (Fig. 7G).

We further evaluated the compensatory effects of STAT4 overexpression in prostate cancer cell lines PC3 and DU145 with reduced IL-11 signalling. In STAT4 overexpression groups (OE STAT4), both PC3 and DU145 cells showed a significant increase in STAT4 expression compared to the control groups (Ctrl), with statistical analysis revealing a p value of less than 0.01 (Figure S1 J). This overexpression was associated with a partial improvement in cell viability in the presence of docetaxel (DTX), as observed in the cell viability assays. Specifically, IC50 values indicated that the OE STAT4 groups in both cell lines maintained higher viability than their respective IL-11 knockdown counterparts (Figures S1 L and M). These results suggest that STAT4 overexpression could mitigate the effects of impaired IL-11 signalling on cell survival in the context of chemotherapeutic stress.

### STAT4 activation and nuclear translocation in response to IL-11 treatment

**Fig. 8:**
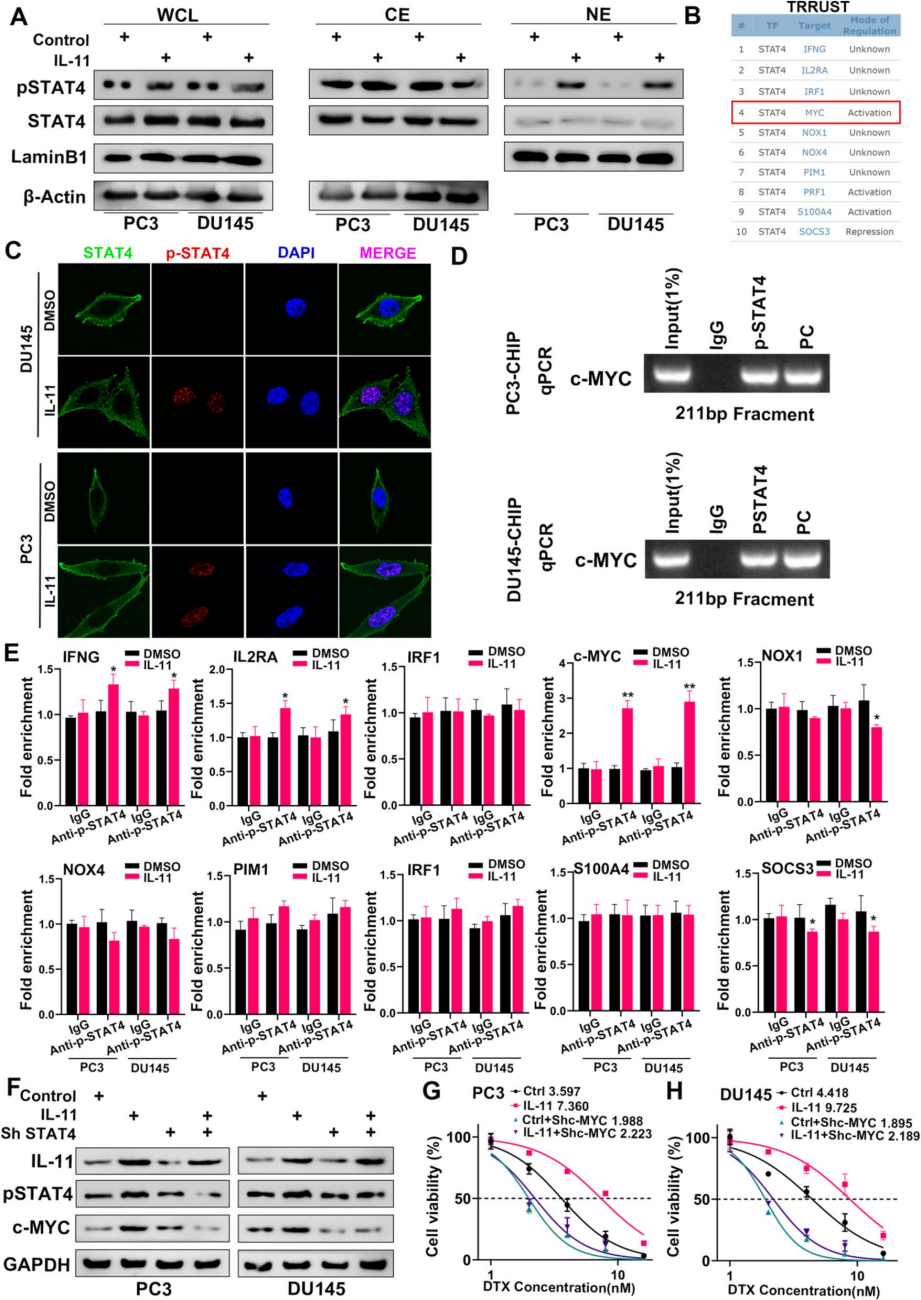
Analysis of STAT4 activation and its downstream effects in PC3 and DU145 cells. **A** Western blot analysis showing phosphorylated STAT4 (pSTAT4) and total STAT4 levels in whole-cell lysates (WCL), cytoplasmic extracts (CE), and nuclear extracts (NE) of PC3 and DU145 cells treated with IL-11. Lamin B1 and β-Actin served as nuclear and cytoplasmic loading controls, respectively. **B** TRRUST database table indicating potential STAT4 target genes and their mode of regulation. **C** Immunofluorescence microscopy images depicting localization of STAT4 and pSTAT4 (red) in DU145 and PC3 cells, with and without IL-11 treatment. DAPI staining (blue) indicates nuclei. **D** Chromatin immunoprecipitation (ChIP) followed by qPCR analysis of c-MYC promoter regions in PC3 and DU145 cells. PCR products were visualized on an agarose gel to confirm the size of the amplicons (211 bp). **E** Quantitative representation of the fold enrichment of STAT4 binding to target gene promoters (IFNG, IL2RA, IRF1, c-MYC, NOX1, PIM1, IRF1, S100A4, SOCS3) in PC3 and DU145 cells, with and without IL-11 treatment. Data are presented as the mean ± SD of triplicate experiments; *p < 0.05, **p < 0.01 compared to IgG control. **F** Western blot analysis to evaluate the effect of STAT4 knockdown (Sh STAT4) on IL-11-induced pSTAT4 and c-MYC expression in PC3 and DU145 cells. GAPDH was used as a loading control. **G** Cell viability assay of PC3 cells with different treatments (control, OE IL-11, STAT4 knockdown, and combined OE IL-11 with STAT4 knockdown) across a range of docetaxel (DTX) concentrations. **H** Similar to (**G**) but showing the results for DU145 cells. The numbers indicate the IC50 values calculated for each treatment condition

In the prostate cancer cell lines PC3 and DU145, IL-11 treatment significantly increased levels of phosphorylated STAT4, indicative of its activation. Western blot analysis demonstrated that pSTAT4 was elevated in both whole-cell lysates and nuclear extracts, suggesting nuclear translocation upon activation (Fig. 8A). Consistently, immunofluorescence microscopy revealed enhanced nuclear localization of pSTAT4 in response to IL-11, with clear nuclear demarcation by DAPI staining (Fig. 8C).

### STAT4 targets gene engagement and the effect of STAT4 knockdown

We utilized the TRRUST database to identify probable target genes of STAT4 and to discern their regulatory patterns. Subsequently, we employed the GeneHancer database (https://genome.ucsc.edu/) to predict transcription factors potentially involved in regulation of these targets. Our analyses indicate a putative regulatory association between STAT4 and c-MYC (Fig. 8B). Chromatin immunoprecipitation followed by quantitative PCR highlighted an IL-11-dependent enrichment of STAT4 at the c-MYC promoter, confirming its role as a direct transcriptional regulator upon cytokine stimulation (Fig. 8D). Quantitative analyses across multiple STAT4 target genes illustrated a significant IL-11-mediated increase in DNA binding, underscoring the transcriptional impact of STAT4 activation (Fig. 8E). Furthermore, STAT4 knockdown reduced both IL-11-induced pSTAT4 and c-MYC expression, validating the functional necessity of STAT4 in the signalling cascade (Fig. 8F).

### Influence of STAT4 modulation on chemotherapeutic sensitivity

Cell viability assays revealed differential sensitivity to docetaxel with IL-11 overexpression and STAT4 knockdown. In PC3 cells, STAT4 suppression decreased cell viability; similar trends were observed in DU145 cells, with calculated IC50 values indicating altered drug responsiveness (Figs. 8G-H). Additionally, the greatest sensitivity to DTX, as indicated by the lowest cell viability, was noted in cells subjected to combined IL-11 knockdown and c-Myc overexpression (Figures S2D, E). These findings suggest that modulating STAT4 or downstream c-MYC activity can influence the therapeutic efficacy of chemotherapeutic agents in prostate cancer. Furthermore, related reports have documented that CBP collaborates with STAT4 in the regulation of transcription [28–31]. Our findings indicate a molecular interaction between CBP and phosphorylated STAT4 (Figure S2F, G). Moreover, we observed that silencing CBP downregulates c-MYC expression and mitigates the decreased sensitivity to docetaxel engendered by IL-11 overexpression (Figure S2H-J).

Our results confirm that the cotranscription factor CBP is implicated in the regulatory control of c-MYC transcription, functioning in conjunction with p-STAT4. This interaction suggests a coordinated regulatory mechanism in which p-STAT4 and CBP may synergistically influence expression of c-MYC and ultimately lead to tumour progression and docetaxel resistance.

## Discussions

The emergent role of cytokine signalling in the context of cancer progression and drug resistance is increasingly being recognized [32–35], with our study contributing key insights into this field. Utilizing single-cell sequencing data from docetaxel-resistant prostate cancer organoids, we identified the exocrine factor IL-11 as being significantly overexpressed in resistant prostate cancer. Further analysis revealed that its downstream receptor, IL-11RA, is predominantly distributed and also significantly overexpressed in the tumor cells themselves.Specifically, our single-cell secretion analysis delineated a mechanism whereby autocrine IL-11 signalling activates the JAK1/STAT4 pathway, leading to phosphorylated STAT4 (pSTAT4) nuclear translocation. In the nucleus, pSTAT4 associates with the cotranscription factor CBP, forming a transcriptional activator complex that enhances c-MYC expression. Notably, in the milieu of prostate cancer, this molecular cascade confers a survival advantage by mediating resistance to the chemotherapeutic agent docetaxel, underscoring the complexity of signalling pathways contributing to therapeutic challenges.

The link between STAT4 activation and c-MYC expression, as revealed by our chromatin immunoprecipitation (ChIP) data, provides compelling evidence of a direct transcriptional regulatory mechanism instrumental in cellular resistance to chemotherapy. Induction of c-MYC is a well-established oncogenic event [35–37], associated with cell growth, proliferation, and survival; thus, its upregulation represents a crucial node in cancer biology. The specificity of pSTAT4's interaction with CBP suggests a finely tuned regulatory system that cancer cells may exploit to acquire resistance against cytotoxic agents. From a therapeutic vantage point, this interaction represents a potential target for novel interventions, and disrupting this complex might restore chemosensitivity.

The interconnection between cytokine signaling and drug resistance has profound therapeutic implications [38–40]. Our findings align with recent studies indicating the crucial role of the IL-6/JAK/STAT3 signaling axis in cancer progression, particularly in influencing the tumor immune microenvironment [41–43]. The potential to overcome resistance by inhibiting the IL-11/IL-11RA/JAK1/STAT4 axis may represent a paradigm shift in prostate cancer treatment. The pursuit of targeted therapies such as JAK1 inhibitors, IL-11 neutralizing antibodies, or molecular disruptors of the pSTAT4-CBP interaction could offer substantial benefits. These approaches, akin to strategies targeting the tumor microenvironment, might resensitize resistant tumors to standard chemotherapies like docetaxel, potentially leading to significant improvements in therapeutic outcomes.

Our research also highlights the role of the tumour microenvironment, which is characterized not only by the cancer cells themselves but also by a complex network of stromal cells, extracellular matrix components, and signalling molecules [44–46]. Within this environment, IL-11 may function not only in an autocrine manner but also in a paracrine manner, affecting adjacent cells and contributing to the creation of a chemoresistant niche. The challenge, therefore, lies in translation of our in vitro findings to the in vivo setting, where the interplay of various cell types and signalling molecules can significantly impact drug response. Animal models that faithfully recapitulate the human tumour microenvironment will be crucial in assessing the therapeutic potential of targeting the IL-11-mediated pathway.

In summary, our study advances our understanding of the mechanistic underpinnings of docetaxel resistance in prostate cancer and underscores potential intervention points for enhancing chemotherapy efficacy. A pivotal aspect of our research was the elucidation of the IL-11/JAK1/STAT4/CBP signaling axis and its role in the upregulation of c-MYC, marking a significant progression in cancer biology. This discovery opens new avenues for exploring the interplay between cytokine signaling and chemoresistance, crucial for the development of next-generation cancer therapies. We acknowledge the significance of incorporating human sample data to substantiate our findings and enhance the clinical relevance. However, we faced considerable challenges in obtaining late-stage, treatment-resistant prostate cancer specimens due to their limited availability. This limitation underscores the need for future work, particularly in vivo studies, which will be instrumental in translating these laboratory findings into clinical strategies that can improve the lives of patients with prostate cancer. By addressing these challenges, subsequent research can bridge the gap between bench and bedside, providing much-needed insights for patient care.

## Conclusion

To sum up,our findings illuminate a crucial link between IL-11 autocrine signalling and docetaxel resistance in prostate cancer via activation of the JAK1/STAT4 pathway. Critical nuclear translocation and binding of pSTAT4 to the cotranscription factor CBP, resulting in the transcriptional amplification of c-MYC, provides a mechanistic understanding of this resistance. These insights not only enhance our knowledge of cytokine-mediated signalling in oncogenesis but also underscore potential therapeutic targets to overcome chemoresistance. Future therapeutic strategies that inhibit this signalling axis may improve the efficacy of docetaxel in treating prostate cancer, indicating a promising direction for subsequent research and clinical intervention.

## Supplementary Information

**Additional file 1: Table S1.** The sequences of small interfering RNAs used in this study

**Additional file 2: Table S2.** Antibodies and used in this study

**Additional file 3: Table S3.** The sequences of primers used in this study

**Additional file 4: Table S4.** List of Abbreviations

**Additional file 5: Figure S1.** (A, D, G) Uniform manifold approximation and projection (UMAP) analysis depicting the cell type clusters identified within the prostate cancer datasets GSE137829, GSE141445, and GSE206962, respectively. Each point represents a single cell, colour-coded according to cell type. (B, E, H) Dot plots showing expression levels of IL11 across different cell types within the prostate cancer microenvironment for datasets GSE137829, GSE141445, and GSE206962, respectively. The y-axis represents the expression level, and each dot represents a single cell. (C, F, I) Dot plots representing expression levels of IL11RA among various cell types in the same datasets as above. Each dot signifies a single cell, and the y-axis corresponds to the expression level of IL11RA. (J) Bar graphs illustrating the relative expression levels of STAT4 in PC3 and DU145 prostate cancer cell lines following overexpression (OE) of STAT4. Data are shown as the mean ± SD; **p < 0.01 indicates statistical significance compared to the control (Ctrl). (L, M) Dose‒response curves showing the viability of PC3 and DU145 cells treated with increasing concentrations of docetaxel (DTX). The cells were subjected to different treatments: control (Ctrl), shRNA-mediated knockdown of IL-11 (Sh IL-11), overexpression of STAT4 (Ctrl + STAT4), and combination of IL-11 knockdown with STAT4 overexpression (Sh IL-11 + STAT4). IC50 values are indicated for each treatment group, demonstrating the impact on cell viability. **Figure S2.** (A) Bar graph representing the fold change in expression of STAT family members in DU145 cells following combined knockdown (KD) of IL-11 and overexpression (OE) of IL-11NC compared to the control. Data are presented as the mean ± SD. (B) Similar to (A), showing fold changes in expression levels of JAK family members in DU145 cells under the same experimental conditions. (C) Forest plot summarizing the hazard ratios from multiple datasets, indicating the association between expression of the IL-11 signature and survival in various patient cohorts. The red line denotes the null effect. (D) Dose‒response curves for PC3 cells treated with docetaxel (DTX) under different conditions: control (Ctrl), IL-11 knockdown (shIL-11), c-MYC overexpression (Ctrl + c-MYC), and combination of IL-11 knockdown with c-MYC overexpression (shIL-11 + c-MYC). IC50 values are indicated for each treatment group. (E) Similar to (D), displaying dose‒response curves and IC50 values for DU145 cells treated with DTX under the same set of conditions. (F) Network diagram illustrating the interactions among various proteins and STAT4, including genetic interactions, posttranslational modifications, and colocalization, based on database and literature evidence. (G) Western blot images from coimmunoprecipitation assays in PC3 and DU145 cells showing the interaction between phosphorylated STAT4 (pSTAT4) and CBP, with IgG as a control. (H) Western blot analysis of PC3 and DU145 cells treated with IL-11 overexpression and shRNA against CBP, assessing the expression of IL-11, CBP, and c-MYC, with GAPDH as a loading control. (I) Cell viability assay for PC3 cells treated with increasing concentrations of DTX, comparing the effects of control, IL-11 overexpression, CBP knockdown, and combined IL-11 treatment with CBP knockdown. (J) Similar to (I), showing cell viability assay results for DU145 cells under the same treatment conditions as PC3 cells. **Figure S3.** (A-G)Scatter plots displaying the log-transformed half maximal inhibitory concentration (IC50) values for various chemotherapeutic agents across different tumour cell clusters (A-E). Each dot represents a single cell, and colours differentiate the clusters. p values are indicated for comparisons between clusters.(H)Comparison of L-11 mRNA expression between PCA tumour and adjacent normal tissues (unpaired t test)(n = 56).(I)Comparison of L-11 mRNA expression between PCA tumour and adjacent normal tissues (paired t test)(n = 56)

## Data Availability

All the data will be provided upon reasonable request.
