# Correction: Single-cell deconvolution algorithms analysis unveils autocrine IL11-mediated resistance to docetaxel in prostate cancer via activation of the JAK1/STAT4 pathway

**DOI:** 10.1186/s13046-024-03257-8

**Published:** 2024-12-28

**Authors:** Bisheng Cheng, Lingfeng Li, Tianlong Luo, Qiong Wang, Yong Luo, Shoumin Bai, Kaiwen Li, Yiming Lai, Hai Huang

**Affiliations:** 1 Department of Urology, Sun Yat-Sen Memorial Hospital, Sun Yat-Sen University, Guangzhou, 510120 China; 2 Guangdong Provincial Key Laboratory of Malignant Tumor Epigenetics and Gene Regulation, Sun Yat-Sen Memorial Hospital, Sun Yat-Sen University, Guangzhou, 510120 China; 3 Guangdong Provincial Clinical Research Center for Urological Diseases, Sun Yat-Sen Memorial Hospital, Sun Yat-Sen University, Guangzhou, 510120 China; 4 Department of Urology, The Sixth Afliated Hospital of Guangzhou Medical University, Qingyuan People’s Hospital, Qingyuan, 511518 Guangdong China; 5 Department of Urology, Nanfang Hospital, Southern Medical University, Guangzhou, 511430 China; 6 Department of Radiation Oncology, Sun Yat-Sen Memorial Hospital, Sun Yat-Sen University, Guangzhou, 510120 China; 7 Department of Urology, Fujian Medical University Union Hospital, Fuzhou, China

**Correction: J Exp Clin Cancer Res 43, 67 (2024)**

**https://doi.org/10.1186/s13046-024-02962-8**

Following the publication of the original article [1], the authors identified an inadvertent duplication of images in Figure 4G due to oversight during manuscript preparation

The correct figure is presented below:

**Incorrect Fig.** 4

**Fig. 4:**
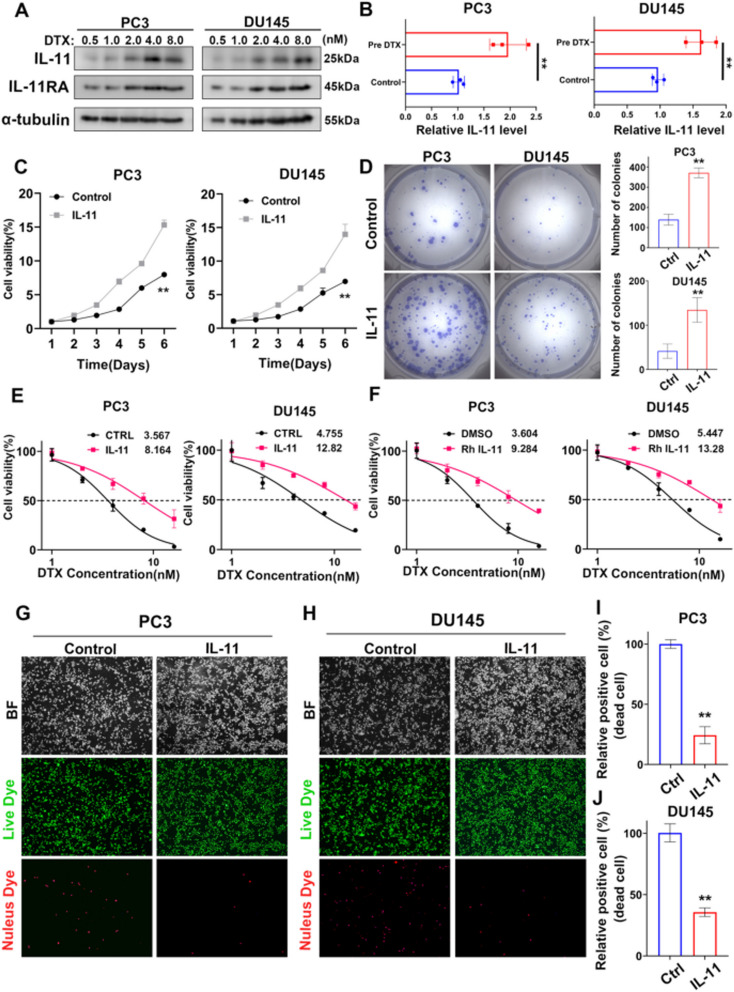
IL-11 contributes to increased viability and chemoresistance in prostate cancer cells. **A** Western blot analyses displaying the dose-dependent expression of IL-11 and IL-11RA in prostate cancer cell lines PC3 and DU145 after treatment with various concentrations of docetaxel (DTX) for 48 h. **B** ELISA measuring IL-11 secretion in the prostate cancer cell lines PC3 and DU145 with or without DTX treatment(1 nM for 48 h). The error bars indicate the standard deviations of three experiments independently. **C** Line graphs representing the percentage of cell viability over a period of 6 days for prostate cancer cell lines PC3 and DU145, comparing the efects of IL-11 overexpression against the control. **D** Images of colony formation in prostate cancer cell lines PC3 and DU145, comparing the control and overexpression treated groups, alongside bar graphs quantifying the number of colonies formed, indicating a signifcant increase in colony formation upon IL-11 treatment. **E** Graphs plotting cell viability percentages against a range of DTX concentrations for prostate cancer cell lines PC3 and DU145, comparing control groups to those treated with IL-11 overexpression.** F** Graphs depicting the efect of recombinant human interleukin 11(RhIL-11) on the cell viability of prostate cancer cell lines PC3 and DU145 at various DTX concentrations compared to the DMSO control. **G**, H Microscopy images of prostate cancer cell lines PC3 (**G**) and DU145 (**H**) displaying live (green) and dead (red) cells in the control and IL-11-treated groups, as indicated by bright feld and fuorescence staining (**I, J**). Bar graphs showing the percentage of dead cells in prostate cancer cell lines PC3 (**I**) and DU145 (**J**), comparing the control and IL-11-treated groups. *P<0.05, **P<0.01, ***P<0.001

**Correct Fig.** 4

**Fig. 4:**
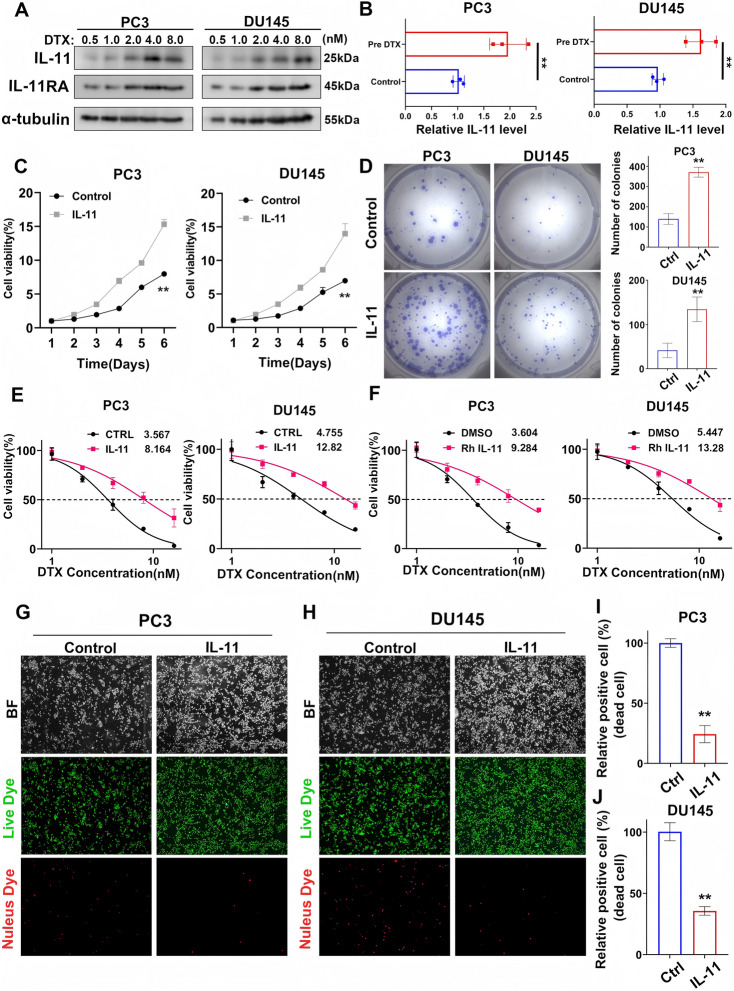
IL-11 contributes to increased viability and chemoresistance in prostate cancer cells. **A** Western blot analyses displaying the dose-dependent expression of IL-11 and IL-11RA in prostate cancer cell lines PC3 and DU145 after treatment with various concentrations of docetaxel (DTX) for 48 h. **B** ELISA measuring IL-11 secretion in the prostate cancer cell lines PC3 and DU145 with or without DTX treatment(1 nM for 48 h). The error bars indicate the standard deviations of three experiments independently. **C** Line graphs representing the percentage of cell viability over a period of 6 days for prostate cancer cell lines PC3 and DU145, comparing the efects of IL-11 overexpression against the control. **D** Images of colony formation in prostate cancer cell lines PC3 and DU145, comparing the control and overexpression treated groups, alongside bar graphs quantifying the number of colonies formed, indicating a signifcant increase in colony formation upon IL-11 treatment. **E** Graphs plotting cell viability percentages against a range of DTX concentrations for prostate cancer cell lines PC3 and DU145, comparing control groups to those treated with IL-11 overexpression.** F** Graphs depicting the efect of recombinant human interleukin 11(RhIL-11) on the cell viability of prostate cancer cell lines PC3 and DU145 at various DTX concentrations compared to the DMSO control. **G**, H Microscopy images of prostate cancer cell lines PC3 (**G**) and DU145 (**H**) displaying live (green) and dead (red) cells in the control and IL-11-treated groups, as indicated by bright feld and fuorescence staining (**I, J**). Bar graphs showing the percentage of dead cells in prostate cancer cell lines PC3 (**I**) and DU145 (**J**), comparing the control and IL-11-treated groups. *P<0.05, **P<0.01, ***P<0.001

The correction does not compromise the validity of the conclusions and the overall content of the article. The original article [1] has been updated.
