# Supplementary material for: Single-cell deconvolution algorithms analysis unveils autocrine IL11-mediated resistance to docetaxel in prostate cancer via activation of the JAK1/STAT4 pathway
Source: J Exp Clin Cancer Res. 2024 Mar 1;43:67. doi: 10.1186/s13046-024-02962-8 (PMC10905933; doi:10.1186/s13046-024-02962-8)
Supplement: Supplementary file 1 — Additional file 1: Table S1. The sequences of small interfering RNAs used in this study [file 13046_2024_2962_MOESM1_ESM.docx]

**Table S1 The sequences of small interfering RNAs used in this study.**

| **NO.** | **Target** | **Sequence 5’-3’** |
| --- | --- | --- |
| **1** | **ShIL-11** | AATTTGTCCCTCAGCTGTGCA |
| **2** | **ShMYC** | TTTAAGGATAACTACCTTGGG |
| **3** | **ShCBP** | AATGATACCTATTCTGATAGC |
| **4** | **ShSTAT4** | ATAATAACTTTGTAGTCTCGC |
