# Supplementary material for: Single-cell deconvolution algorithms analysis unveils autocrine IL11-mediated resistance to docetaxel in prostate cancer via activation of the JAK1/STAT4 pathway
Source: J Exp Clin Cancer Res. 2024 Mar 1;43:67. doi: 10.1186/s13046-024-02962-8 (PMC10905933; doi:10.1186/s13046-024-02962-8)
Supplement: Supplementary file 2 — Additional file 2: Table S2. Antibodies and used in this study [file 13046_2024_2962_MOESM2_ESM.docx]

**Table S2. Antibodies and used in this study.**

| **Application** | **Protein or histone modification** | **Manufacturer** | **Cat number** | **Dilution for usage** |
| --- | --- | --- | --- | --- |
| Western blotting | IL-11 | Proteintech | 55169-1-AP | 1:2000 |
| Western blotting | IL-11RA | Proteintech | 10264-1-AP | 1:1000 |
| Western blotting | JAK1 | CST | #3344 | 1:1000 |
| Western blotting | p-JAK1 | CST | #74129 | 1:1000 |
| Western blotting | STAT4 | CST | #2653 | 1:1000 |
| Western blotting | p-STAT4 | CST | #4134 | 1:1000 |
| Western blotting | C-MYC | CST | #18583 | 1:1000 |
| Western blotting | CBP | CST | #7389 | 1:1000 |
| Western blotting | α-tubulin | Proteintech | 11224-1-AP | 1:5000 |
| Western blotting | GAPDH | Proteintech | 10494-1-AP | 1:3000 |
| Immunohistochemistry | IL-11 | Proteintech | 55169-1-AP | 1:200 |
| Immunohistochemistry | Ki-67 | Proteintech | 27309-1-AP | 1:200 |
| Co - immunoprecipitation | CBP | CST | #7389 | 1:50 |
| Co - immunoprecipitation | p-STAT4 | CST | #4134 | 1:50 |
| Co - immunoprecipitation | IgG | CST | #3900 | 1:500 |
| CHIP | p-STAT4 | CST | #4134 | 1:50 |
| Neutralising | IL-11 | Abcam | ab89887 |  |
| Elisa | IL-11 | Abcam | ab100551 |  |
| IL-11 antagonist | Soluble IL-11Rα | Abcam | ab132575 |  |

Proteintech (Wuhan, Hubei, China). CST (Danvers, Massachusetts, USA).Abcam (Cambridge, UK)
