# Supplementary material for: Single-cell deconvolution algorithms analysis unveils autocrine IL11-mediated resistance to docetaxel in prostate cancer via activation of the JAK1/STAT4 pathway
Source: J Exp Clin Cancer Res. 2024 Mar 1;43:67. doi: 10.1186/s13046-024-02962-8 (PMC10905933; doi:10.1186/s13046-024-02962-8)
Supplement: Supplementary file 3 — Additional file 3: Table S3. The sequences of primers used in this study [file 13046_2024_2962_MOESM3_ESM.docx]

**Table S3 The sequences of primers used in this study.**

| **NO.** | **Target** | **Forward (5'-3')** | **Reverse (5'-3')** | |
| --- | --- | --- | --- | --- |
| **1** | **IL-11** | GGACCACAACCTGGATTCCCTG | AGTAGGTCCGCTCGCAGCCTT |  |
| **2** | **IL-11RA** | CTGGGCTAGGGCATGAACTG | CTGGGACTCCAAGTGCAAGA |  |
| **3** | **c-MYC** | TCGCTGCTGTCCTCCGAGTCC | GGTTTGCCTCTTCTCCACAGAC |  |
| **4** | **CBP** | CACCATCTGTGGCTACTCCTCA | GGTTTCAGCACTGGTCACAGAG |  |
| **5** | **JAK1** | GAGACAGGTCTCCCACAAACAC | GTGGTAAGGACATCGCTTTTCCG |  |
| **6** | **STAT4** | CAGTGAAAGCCATCTCGGAGGA | TGTAGTCTCGCAGGATGTCAGC |  |
| **7** | **GAPDH** | CAAGGCTGAGAACGGGAAG | TGAAGACGCCAGTGGACTC |  |
