# Supplementary material for: Single-cell deconvolution algorithms analysis unveils autocrine IL11-mediated resistance to docetaxel in prostate cancer via activation of the JAK1/STAT4 pathway
Source: J Exp Clin Cancer Res. 2024 Mar 1;43:67. doi: 10.1186/s13046-024-02962-8 (PMC10905933; doi:10.1186/s13046-024-02962-8)
Supplement: Supplementary file 4 — Additional file 4: Table S4. List of Abbreviations [file 13046_2024_2962_MOESM4_ESM.docx]

**Table S4 List of Abbreviations.**

| **Full name** | **Abbreviation** |
| --- | --- |
| interleukin-11 | IL-11 |
| interleukin-11 receptor subunit alpha | IL-11RA |
| CREB-binding protein | CBP |
| American Type Culture Collection | ATCC |
| short tandem repeat | STR |
| Immunohistochemistry | IHC |
| quantitative real-time polymerase chain reaction | qPCR |
| short hairpin RNA | shRNA |
| dimethyl sulfoxide | DMSO |
| prostate cancer | PCa |
| The Cancer Genome Atlas | TCGA |
| recurrence-free survival | RFS |
| overall survival | OS |
| differentially expressed genes | DEGs |
| Gene Ontology | GO |
| Kyoto Encyclopedia of Genes and Genomes | KEGG |
| docetaxel | DTX |
| recombinant human IL-11 | Rh IL-11 |
| IL-11 neutralizing antibody | IL-11 Neu |
| IL-11 antagonist | Anti IL-11RA |
| Control | Ctrl |
| Gene set enrichment analysis | GSEA |
| Uniform manifold approximation and projection | UMAP |
| knockdown | KD |
| overexpression | OE |
