# Supplementary material for: Single-cell deconvolution algorithms analysis unveils autocrine IL11-mediated resistance to docetaxel in prostate cancer via activation of the JAK1/STAT4 pathway
Source: J Exp Clin Cancer Res. 2024 Mar 1;43:67. doi: 10.1186/s13046-024-02962-8 (PMC10905933; doi:10.1186/s13046-024-02962-8)
Supplement: Supplementary file 5 — Additional file 5: Figure S1. (A, D, G) Uniform manifold approximation and projection (UMAP) analysis depicting the cell type clusters identified within the prostate cancer datasets GSE137829, GSE141445, and GSE206962, respectively. Each point represents a single cell, colour-coded according to cell type. (B, E, H) Dot plots showing expression levels of IL11 across different cell types within the prostate cancer microenvironment for datasets GSE137829, GSE141445, and GSE206962, respectively. The y-axis represents the expression level, and each dot represents a single cell. (C, F, I) Dot plots representing expression levels of IL11RA among various cell types in the same datasets as above. Each dot signifies a single cell, and the y-axis corresponds to the expression level of IL11RA. (J) Bar graphs illustrating the relative expression levels of STAT4 in PC3 and DU145 prostate cancer cell lines following overexpression (OE) of STAT4. Data are shown as the mean ± SD; **p < 0.01 indicates statistical significance compared to the control (Ctrl). (L, M) Dose‒response curves showing the viability of PC3 and DU145 cells treated with increasing concentrations of docetaxel (DTX). The cells were subjected to different treatments: control (Ctrl), shRNA-mediated knockdown of IL-11 (Sh IL-11), overexpression of STAT4 (Ctrl + STAT4), and combination of IL-11 knockdown with STAT4 overexpression (Sh IL-11 + STAT4). IC50 values are indicated for each treatment group, demonstrating the impact on cell viability. Figure S2. (A) Bar graph representing the fold change in expression of STAT family members in DU145 cells following combined knockdown (KD) of IL-11 and overexpression (OE) of IL-11NC compared to the control. Data are presented as the mean ± SD. (B) Similar to (A), showing fold changes in expression levels of JAK family members in DU145 cells under the same experimental conditions. (C) Forest plot summarizing the hazard ratios from multiple datasets, indicating the association b [file 13046_2024_2962_MOESM5_ESM.docx]

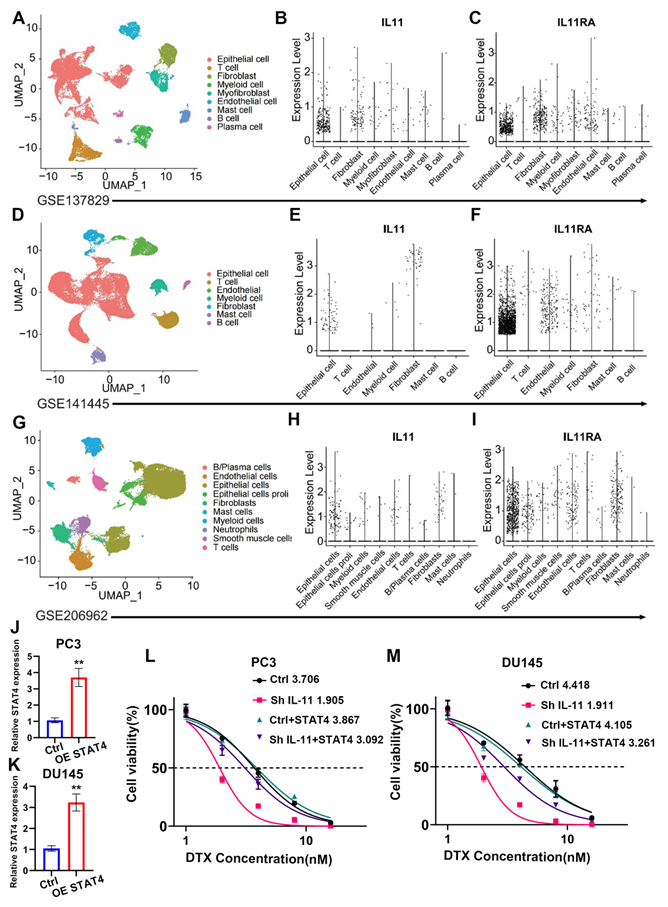


**Figure S1.**

(A, D, G) Uniform manifold approximation and projection (UMAP) analysis depicting the cell type clusters identified within the prostate cancer datasets GSE137829, GSE141445, and GSE206962, respectively. Each point represents a single cell, colour-coded according to cell type. (B, E, H) Dot plots showing expression levels of IL11 across different cell types within the prostate cancer microenvironment for datasets GSE137829, GSE141445, and GSE206962, respectively. The y-axis represents the expression level, and each dot represents a single cell. (C, F, I) Dot plots representing expression levels of IL11RA among various cell types in the same datasets as above. Each dot signifies a single cell, and the y-axis corresponds to the expression level of IL11RA. (J) Bar graphs illustrating the relative expression levels of STAT4 in PC3 and DU145 prostate cancer cell lines following overexpression (OE) of STAT4. Data are shown as the mean ± SD; **p<0.01 indicates statistical significance compared to the control (Ctrl). (L, M) Dose‒response curves showing the viability of PC3 and DU145 cells treated with increasing concentrations of docetaxel (DTX). The cells were subjected to different treatments: control (Ctrl), shRNA-mediated knockdown of IL-11 (Sh IL-11), overexpression of STAT4 (Ctrl+STAT4), and combination of IL-11 knockdown with STAT4 overexpression (Sh IL-11+STAT4). IC50 values are indicated for each treatment group, demonstrating the impact on cell viability.


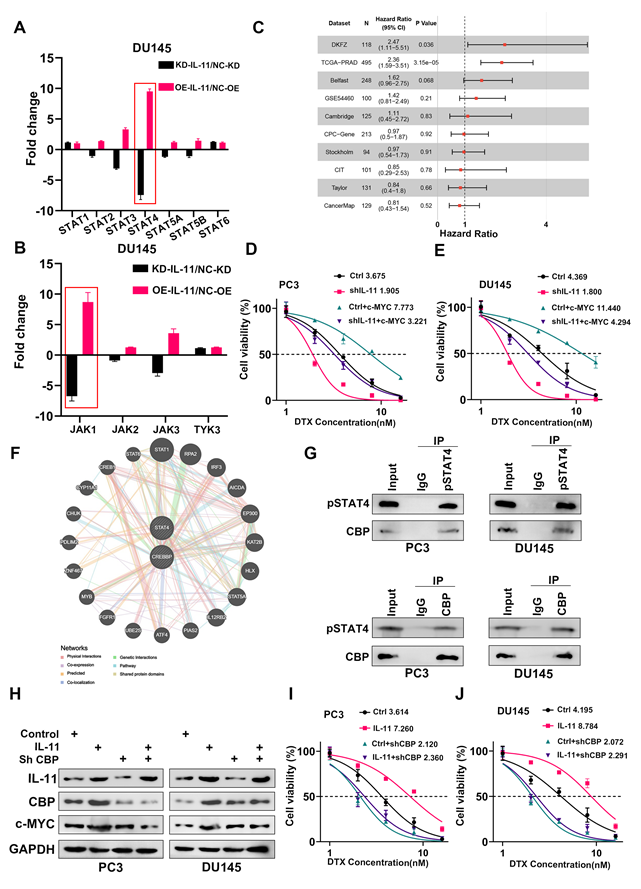


**Figure S2.**

(A) Bar graph representing the fold change in expression of STAT family members in DU145 cells following combined knockdown (KD) of IL-11 and overexpression (OE) of IL-11NC compared to the control. Data are presented as the mean ± SD. (B) Similar to (A), showing fold changes in expression levels of JAK family members in DU145 cells under the same experimental conditions. (C) Forest plot summarizing the hazard ratios from multiple datasets, indicating the association between expression of the IL-11 signature and survival in various patient cohorts. The red line denotes the null effect. (D) Dose‒response curves for PC3 cells treated with docetaxel (DTX) under different conditions: control (Ctrl), IL-11 knockdown (shIL-11), c-MYC overexpression (Ctrl+c-MYC), and combination of IL-11 knockdown with c-MYC overexpression (shIL-11+c-MYC). IC50 values are indicated for each treatment group. (E) Similar to (D), displaying dose‒response curves and IC50 values for DU145 cells treated with DTX under the same set of conditions. (F) Network diagram illustrating the interactions among various proteins and STAT4, including genetic interactions, posttranslational modifications, and colocalization, based on database and literature evidence. (G) Western blot images from coimmunoprecipitation assays in PC3 and DU145 cells showing the interaction between phosphorylated STAT4 (pSTAT4) and CBP, with IgG as a control. (H) Western blot analysis of PC3 and DU145 cells treated with IL-11 overexpression and shRNA against CBP, assessing the expression of IL-11, CBP, and c-MYC, with GAPDH as a loading control. (I) Cell viability assay for PC3 cells treated with increasing concentrations of DTX, comparing the effects of control, IL-11 overexpression, CBP knockdown, and combined IL-11 treatment with CBP knockdown. (J) Similar to (I), showing cell viability assay results for DU145 cells under the same treatment conditions as PC3 cells.


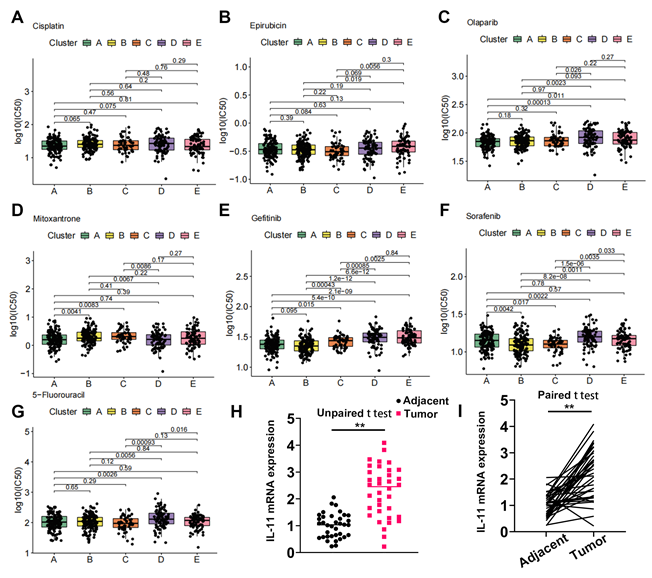


**Figure S3.**

(A-G)Scatter plots displaying the log-transformed half maximal inhibitory concentration (IC50) values for various chemotherapeutic agents across different tumour cell clusters (A-E). Each dot represents a single cell, and colours differentiate the clusters. *p* values are indicated for comparisons between clusters.(H)Comparison of L-11 mRNA expression between PCA tumour and adjacent normal tissues (unpaired t test)(n=56).(I)Comparison of L-11 mRNA expression between PCA tumour and adjacent normal tissues (paired t test)(n=56).
